# Efficacy and Safety of Amphotericin B Emulsion versus Liposomal Formulation in Indian Patients with Visceral Leishmaniasis: A Randomized, Open-Label Study

**DOI:** 10.1371/journal.pntd.0003169

**Published:** 2014-09-18

**Authors:** Shyam Sundar, Krishna Pandey, Chandreshwar Prasad Thakur, Tara Kant Jha, Vidya Nand Ravi Das, Neena Verma, Chandra Shekhar Lal, Deepak Verma, Shahnawaz Alam, Pradeep Das

**Affiliations:** 1 Department of Medicine, Institute of Medical Sciences, Banaras Hindu University, Varanasi, India; 2 Department of Clinical Medicine, Rajendra Memorial Research Institute of Medical Sciences (Indian Council of Medical Research), Agamkuan, Patna, Bihar, India; 3 Balaji Utthan Sansthan, Kala-azar Research Centre, Patna, Bihar, India; 4 Kalazar Research Centre, Brahmpura, Muzaffarpur, Bihar, India; 5 Department of Pathology, Rajendra Memorial Research Institute of Medical Sciences (Indian Council of Medical Research), Agamkuan, Patna, Bihar, India; 6 Department of Biochemistry, Rajendra Memorial Research Institute of Medical Sciences (Indian Council of Medical Research), Agamkuan, Patna, Bihar, India; 7 Kala-azar Medical Research Center, Muzaffarpur, Bihar, India; 8 Rajendra Memorial Research Institute of Medical Sciences (Indian Council of Medical Research), Agamkuan, Patna, Bihar, India; New York University, United States of America

## Abstract

**Background:**

India is home to 60% of the total global visceral leishmaniasis (VL) population. Use of long-term oral (e.g. miltefosine) and parenteral drugs, considered the mainstay for treatment of VL, is now faced with increased resistance, decreased efficacy, low compliance and safety issues. The authors evaluated the efficacy and safety of an alternate treatment option, i.e. single infusion of preformed amphotericin B (AmB) lipid emulsion (ABLE) in comparison with that of liposomal formulation (LAmB).

**Methods:**

In this multicentric, open-label study, 500 patients with VL were randomly assigned in a 3∶1 ratio to receive 15 mg/kg single infusion of either ABLE (N = 376) or LAmB (N = 124). Initial cure (Day 30/45), clinical improvement (Day 30) and long term definitive cure (Day 180) were assessed.

**Findings:**

A total of 326 (86.7%) patients in the ABLE group and 122 (98.4%) patients in the LAmB group completed the study. Initial cure was achieved by 95.9% of patients in the ABLE group compared to 100% in the LAmB group (p = 0.028; 95% CI: −0.0663, −0.0150). Clinical improvement was comparable between treatments (ABLE: 98.9% vs. LAmB: 98.4%). Definitive cure was achieved in 85.9% with ABLE compared to 98.4% with LAmB. Infusion-related pyrexia (37.2% vs. 32.3%) and chills (18.4% vs. 18.5%) were comparable between ABLE and LAmB, respectively. Treatment-related serious adverse events were fewer in ABLE (0.3%) compared to LAmB (1.6%). Two deaths occurred in the ABLE group, of which one was probably related to the study drug. Nephrotoxicity and hepatotoxicity was not observed in either group.

**Conclusions:**

ABLE 15 mg/kg single infusion had favorable efficacy and was well tolerated. Considering the demographic profile of the population in this region, a single dose treatment offers advantages in terms of compliance, cost and applicability.

**Trial Registration:**

www.clinicaltrials.gov NCT00876824

## Author Summary

Visceral leishmaniasis (VL) is highly prevalent in northeastern India, particularly the state of Bihar and its bordering areas with Bangladesh and Nepal. The current standards of treatment, namely, miltefosine (oral) and pentavalent antimonials (parenteral) have long treatment durations and are faced with increasing resistance, decreased efficacy, low compliance and safety issues. In this regard, lipid formulations of amphotericin B (AmB) have become an attractive treatment option due to their high efficacy, shorter treatment regimens and favorable safety profiles. This Phase III study evaluated the efficacy and safety of preformed AmB lipid emulsion (ABLE) versus liposomal AmB (LAmB) (both 15 mg/kg single dose infusions) in the treatment of VL. ABLE showed favorable efficacy measured in terms of initial cure at Day 30/45, and overall clinical improvement. ABLE was well tolerated and its adverse event profile was consistent with previously documented findings. Based on the favorable efficacy and safety profile of ABLE, and considering the demographic profile of the population in the endemic region, a single dose treatment may offer advantages in terms of compliance, cost and applicability.

## Introduction

Visceral leishmaniasis (VL), also known as kala-azar, is a vector-borne disease transmitted to humans by the bite of an infected sandfly [1]. Globally, around 200,000–400,000 cases of VL occur each year of which 60% cases occur in India alone [2]. Kala-azar is a major public health problem in the areas of its prevalence, principally India and its neighbors Bangladesh and Nepal. In India, the disease is highly prevalent in Bihar, Jharkhand, West Bengal and pockets of eastern Uttar Pradesh. Among these, Bihar is the most affected with >90% of cases [3], of which 10% are fatal [2].

Contrary to the severity, few drugs are available for its treatment and are further limited by safety, reduced effectiveness and challenges in administration. Use of pentavalent antimonials, the mainstay of treatment for over 70 years, has been limited by its resistance and toxicities [4]. In India, almost 65% of previously untreated cases fail to respond promptly or relapse after treatment with antimonials [5]. Efficacy of the first-line oral treatment, miltefosine (MF) has declined rapidly over the past decade (final cure rate: 96.7% in 1999, 94% in 2002, 82% in 2007, and 72% in 2011) and is also associated with gastrointestinal side effects [6]–[9]. In addition, owing to its teratogenic effects, treatment with MF may require strict medical monitoring for treating women of child bearing age, which considering current demographic outlook of India is a significant factor [10], [11]. Paramomycin, an aminoglycoside, had shown 94% cure rate but is associated with systemic hepatic toxicity; the current regimen of 21 daily injections is also a major disadvantage for routine clinical use [12].

Amphotericin B (AmB), currently a second line drug used for treatment of VL, is highly effective with cure rates of 97%; however, the administration of 15 intravenous injections (i.v.) over 30 days of hospitalization, coupled with infusion- and drug-related adverse effects [13], has limited its wide-spread use. Liposomal formulations of AmB (LAmB) are better tolerated and thus preferable to conventional AmB [14], [15]. Despite the WHO-negotiated price of LAmB, treatment with it still remains limited and unaffordable in India [16]. Educational, social and economic background of patients in endemic areas entails therapy that does not bother patients with cost, undue compliance issues and long treatment duration, making a simplified treatment regimen a need of the hour. Thus, an affordable premixed AmB deoxycholate with lipid emulsion (ABLE) was developed (licensed in India) [17] and can be a potential candidate for treatment and elimination in endemic countries. Previous Phase II studies have reported safety and efficacy with a single infusion of 15 mg/kg of ABLE [17], [18] in the treatment of VL.

This Phase III study was conducted to evaluate the efficacy and safety of ABLE versus LAmB (both 15 mg/kg single dose infusions) in the treatment of VL.

## Methods

### Ethics statement

The protocol was approved by an Independent Ethics Committee or Institutional Review Board at each study site and the study was conducted in accordance with the ethical principles originating in the Declaration of Helsinki and in accordance with ICH Good Clinical Practice guidelines, applicable regulatory requirements, and in compliance with the protocol. All participants including guardians in case of minors provided written informed consent to participate in the study. This study was registered at ClinicalTrials.gov (NCT00876824).

### Study design

This was a prospective, multicentric, randomized, open-label, comparative Phase III study.

### Participants

Patients were enrolled from 4 centers in Bihar, India, between August 2009 and January 2011. Male and female, aged 5–65 years (both inclusive) diagnosed with VL (fever >2 weeks duration and splenomegaly), who had amastigotes (*Leishmania donovani* bodies) at prescreening (detected by recombinant K39 protein [rK39] dipstick test) and confirmed VL by splenic or bone marrow aspirate smear examination were included in the study. Other inclusion criteria were hemoglobin (Hb) ≥5 g/dL, white blood cells count ≥1000/cmm, platelet count ≥50000/cmm, prothrombin time ≤4 seconds above the control, and alkaline transaminase, aspartate transaminase, and alkaline phosphatase ≤2.5 times the upper limit of normal. Patients with past history of treatment with AmB or any other drug for VL within 30 days prior to screening, major surgery within 2 weeks prior to screening, concurrent malaria, alcoholism or illicit drug use/abuse or any condition associated with poor compliance, hypersensitivity to AmB, inactive ingredients of ABLE and LAmB formulations were excluded from the study. Patients who received any of the prohibited medications (any other investigational drugs, antileishmanial drugs other than study drug, corticosteroids, skeletal muscle relaxants, cyclosporine, digoxin, vancomycin, aminoglycosides, antifungal, immunosuppresive agents, and all potentially nephrotoxic drugs), who were positive for human immunodeficiency virus, hepatitis C virus and hepatitis B surface antigen infections and immune-compromised, were also excluded from the study.

### Interventions

**Figure 1:**
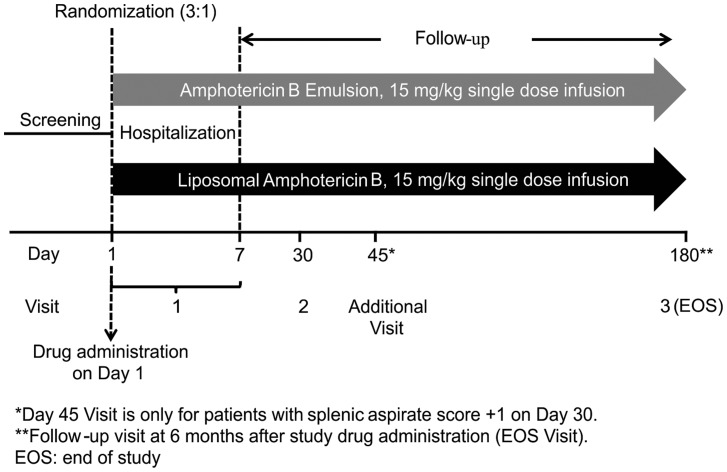
Study design.

Eligible patients were randomized (3∶1) to receive either ABLE or LAmB, as 15 mg/kg single dose infusions (Figure 1). Prior to administration of full-dose, patients received initial test doses (ABLE and LAmB) of 1 mg in 5% dextrose as an infusion over ∼15–20 minutes for the ABLE treatment and over a period of 10 minutes for the LAmB treatment. Patients who experience any hypersensitivity or cardiopulmonary complications of hypersensitivity were withdrawn from the study. Full dose of ABLE and LAmB was diluted in 5% dextrose to a concentration of 1 mg/ml prior to administration. Patients received full doses of respective treatment in single intravenous infusion over 4–6 hours. Premedication was not allowed prior to the study drug administration. Patients were hospitalized for 7 days starting from day of first dose of the study drug for safety and efficacy evaluation.

### Outcome measures

To assess parasitological cure, splenic aspirate (or bone marrow aspirate in whom splenic aspirates was not feasible) was performed on Day 30 post infusion. Parasite density was graded by microscopy using a conventional logarithmic scale of 0 (no amastigotes/1000 oil-immersion fields) to +6 (>100 amastigotes/1000 oil-immersion field). Patients with +1 score on Day 30 were re-evaluated on Day 45. Patients were considered to achieve initial cure if the score was 0 on either Day 30 or 45. Patients with score >+1 on Day 30 and/or ≥1 on Day 45 were considered as treatment failures. They were withdrawn from the study and treated with rescue medication in appropriate doses as indicated in the protocol (LAmB 5 mg/kg i.v. on Days 1, 3, 5 and 7 or alternative antileishmanial drug in appropriate doses). Patients were further observed for clinical improvement presented as absence of fever and one or more of the following: increase in Hb concentration by ≥10%, weight gain, or decrease in spleen size by ≥33% (compared from baseline to Day 30). Patients who had achieved initial cure were followed-up for 6 months to study any sign/symptoms of relapse of VL. Patients with an initial cure and no signs or symptoms of VL at the last visit were considered to have achieved definitive cure.

### Other data collection

All the patients were monitored for incidence of infusion related toxicities, nephrotoxicity, hepatotoxicity, number of adverse events (AEs), treatment-emergent AEs (TEAEs), serious AEs (SAEs), and laboratory values (normal; abnormal, not clinically significant; and abnormal, clinically significant) for different parameters. Any drug related Grade III or higher AEs recorded for abnormal clinically significant renal function tests or liver function tests were classified as nephrotoxicity or hepatotoxicity as per National Cancer Institute Common Terminology Criteria (NCI-CTC) AE, version 3.

### Sample size calculation

A total of 500 patients in a 3∶1 ratio (375 in ABLE and 125 in LAmB) were planned to be enrolled assuming a dropout rate of 20% and non-inferiority margin fixed at −0.10. This was expected to provide an estimated difference in proportions of patients achieving definitive cure for ABLE vs. LAmB equals to zero, with at least 80% power for the non-inferiority test.

### Randomization

The permuted block randomization, with block size of 4, and ratio of 3∶1 in the two groups (ABLE and LAmB) were generated for each center. Eligible patients were sequentially allotted to unique subject ID and treatment (ABLE or LAmB) as per randomization schedule for that center. The screening and randomization log was maintained.

### Statistical methods

Data were expressed as means (±SD) for continuous variables and percentages for categorical variables. Proportion of patients achieving all three-efficacy (initial cure, clinical improvement and definitive cure) endpoints were to be compared across the two treatment groups.

For initial cure and clinical improvement, the data was to be analyzed using chi-square test at 5% level of significance. But as the expected number of patients achieving or non achieving initial cure in any of the treatment group was found to be <5, a Fisher's exact test was used. P-value<0.05 was considered as statistically significant. For definitive cure, non-inferiority was assessed by looking at the lower end of a two-sided 95% confidence interval (CI) of the difference P_test_ - P_ref_ (the difference in the proportions of patients achieving definitive cure in ABLE (P_test_) and LAmB (P_ref_). Non-inferiority was only accepted if the lower limit of the two-sided 95% CI was greater than the non-inferiority margin of −0.10. For the three efficacy parameters, the 95% CI was calculated by using Wald's confidence interval with Yate's continuity correction formula.

For safety, the number and percentage of patients experiencing toxicities and AEs (including laboratory abnormalities) across two treatment groups were summarized. Percentages were based on total number of patients in ITT population in each treatment groups.

The efficacy analysis was performed on modified intent-to-treat (mITT) population, which includes all patients who received study drug as per the protocol specified duration and had at least one efficacy assessment throughout the study. Safety analysis was performed on intent-to-treat (ITT) population, which includes all patients who received the treatment of study drug.

## Results

### Patient population

**Figure 2:**
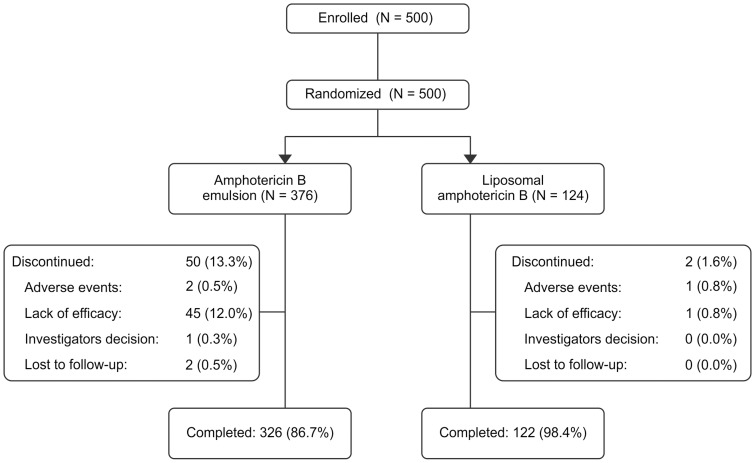
CONSORT patient flowchart.

Of the 500 patients randomized, 376 patients received ABLE and 124 patients received LAmB. The percentage of patients who completed the study was lower in the ABLE group (86.7%) compared with the LAmB group (98.4%). A total of 50 (13.3%) patients discontinued the study in the ABLE group compared to 2 patients (1.6%) in the LAmB group (Figure 2). Patients were predominantly men (60.8%); mean age was 24.8 years (range: 5 to 62 years), and the rest of the baseline characteristics were similar for both groups (Table 1). In this study, all patients were qualified for treatment and included in the ITT population.

**Table 1:** Demographic and baseline characteristics (ITT population).

		Amphotericin B		
Characteristic		Lipid emulsion (N = 376)	Liposomal formulation (N = 124)	Overall (N = 500)
**Age at onset, years**		24.3±14.26	26.3±15.23	24.8±14.52
**Sex, n (%)**	Women	148 (39.4)	48 (38.7)	196 (39.2)
	Men	228 (60.6)	76 (61.3)	304 (60.8)
**Height (cm)**		144.51±19.45	145.75±19.08	144.81±19.35
**Weight (kg)**		37.10±14.71	39.09±15.26	37.59±14.86
**Spleen size (cm)**		6.06±3.92	6.31±3.94	6.13±3.92
**Hemoglobin (g/dL)**	Normal, n (%)	8 (2.1)	4 (3.2)	12 (2.4)
	Abnormal, NCS, n (%)	368 (97.9)	120 (96.8)	488 (97.6)
	Abnormal, CS, n (%)	0 (0.0)	0 (0.0)	0 (0.0)
**White blood cells (/µL)**	Normal, n (%)	111 (29.5)	47 (37.9)	158 (31.6)
	Abnormal, NCS, n (%)	265 (70.5)	77 (62.1)	342 (68.4)
	Abnormal, CS, n (%)	0 (0.0)	0 (0.0)	0 (0.0)
**Platelets (/µL)**	Normal, n (%)	160 (42.6)	56 (45.2)	216 (43.2)
	Abnormal, NCS, n (%)	216 (57.4)	68 (54.8)	284 (56.8)
	Abnormal, CS, n (%)	0 (0.0)	0 (0.0)	0 (0.0)
**Creatinine (mg/dL)**		0.83±0.23	0.83±0.22	0.83±0.23

Data are mean ± standard deviation, unless stated; ITT = Intent-to-Treat; NCS = not clinically significant; CS = clinically significant.

### Efficacy

#### Initial cure

Achievement of initial cure (on Day 30 and/or Day 45) was 95.9% in the ABLE group compared to 100% in the LAmB group (p = 0.028; 95% CI: −0.0663, −0.0150) (Table 2).

**Table 2:** Proportion of patients achieving initial and definitive cure after treatment with amphotericin B emulsion and liposomal amphotericin B (mITT population).

	Amphotericin B		
	Lipid emulsion (N = 369)	Liposomal formulation (N = 122)	P (95% CI)
**Initial cure**	354 (95.9)	122 (100.0)	0.0280 (−0.0663, −0.0150)
**Clinical improvement**	365 (98.9)	120 (98.4)	0.6414 (−0.0248, 0.0359)
**Definitive cure**	317 (85.9)	120 (98.4)	(−0.1720, −0.0770)

Data are n (%); mITT = modified Intent-to-Treat; CI = confidence interval.

#### Clinical improvement

**Figure 3:**
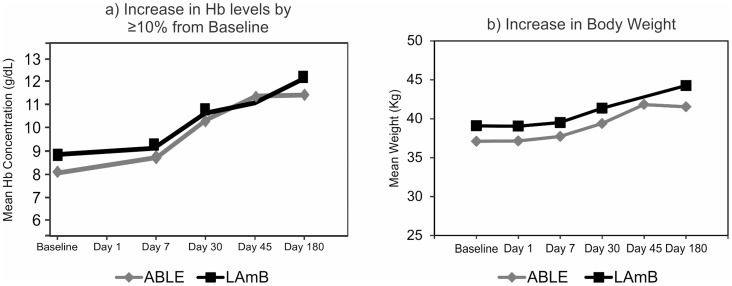
Increase in Hb levels (A) and body weight (B) from baseline after treatment with lipid and liposomal formulations of amphotericin B (mITT population).

During evaluation of clinical improvement at Day 30, no patients in the ABLE and LAmB groups had fever. Hb concentration was increased by at least 10% from baseline in 293 (79.4%) patients treated with ABLE and 80 (65.6%) patients treated with LAmB (Figure 3a). Weight gain was observed in 318 (86.2%) patients in the ABLE group and 103 (84.4%) patients in the LAmB group (Figure 3b). Similarly spleen size had decreased by at least 33% compared to baseline in 348 (94.3%) patients in the ABLE and 117 (95.9%) patients in the LAmB group. Overall, clinical improvement was 98.9% in ABLE group compared to 98.4% in LAmB group (p = 0.6414; 95% CI: −0.0248, 0.0359) (Table 2).

#### Definitive cure

The proportion of patients with no clinical signs and symptoms of relapse of kala-azar for 6 months follow-up was 85.9% in the ABLE group compared to 98.4% in the LAmB group (95% CI [−0.1720, −0.0770]).

### Safety

The proportion of patients with at least 1 AE was comparable in both the ABLE group and in the LAmB group (202 [53.7%] and 61 [49.2%]) (Table 3). The majority of AEs considered to be possibly related to the study drug was similar in both treatment groups (45.2%). Similarly, TEAEs in the ABLE (179 [47.6%]) and LAmB (56 [45.2%]) were comparable. The most common TEAEs in both ABLE and LAmB were chills (18.4% and 18.5%) and pyrexia (37.2% and 32.3%), respectively (Table 3). The majority (>35%) of the patients (152 [40.4%] vs. 46 [37.1%]) experienced AEs of mild intensity.

**Table 3:** Summary of adverse events (AEs) and treatment-emergent adverse events (TEAEs) (ITT population).

		Amphotericin B		
		Lipid Emulsion, N = 376	Liposomal Formulation, N = 124	Overall N = 500
**Summary of AEs**	Patients with ≥1 AE	202 (53.7)	61(49.2)	263(52.6)
	Patients with ≥1 SAE	2 (0.5)	2 (1.6)	4 (0.8)
	Patients with ≥1 TEAEs	179 (47.6)	56 (45.2)	235 (47.0)
	Patients with TESAEs	1 (0.3)	2 (1.6)	3 (0.6)
	AEs leading to death	2 (0.5)	0 (0.0)	2 (0.4)
	AEs leading to permanent interruption of study drug	0 (0.0)	1 (0.8)	1 (0.2)
**TEAEs observed in ≥2% of patients**	Pyrexia	125 (33.2)	37 (29.8)	162 (32.4)
	Chills	68 (18.1)	22 (17.7)	90 (18.0)
	Vomiting	5 (1.3)	3 (2.4)	8 (1.6)
	Diarrhea	1 (0.3)	3 (2.4)	4 (0.8)

Data are n (%); ITT = Intent-to-Treat; SAE = serious adverse events; TESAEs = treatment-emergent serious adverse events.

Two patients in each group had at least one SAE (ABLE 0.5% vs. LAmB 1.6%). Of these SAEs, one patient (0.3%) in the ABLE and 2 (1.6%) patients in the LAmB group was considered treatment-related. The SAEs that occurred in ABLE were anemia, diarrhea, vomiting and sudden death; while in LAmB, pancytopenia and diarrhea (in one patient each). In total, two deaths occurred in the ABLE group due to AEs. One death occurred 2 days after drug administration due to severe diarrhea and was considered probably related to the drug. The other death occurred on Day 157 and was not related to the study drug. In the LAmB group, one patient (0.8%) was discontinued from the study due to urticaria (Table 3).

The incidence of infusion related toxicities on Day 1 was comparable between the groups (43.6% in ABLE and 41.9% in LAmB group). None of the patients in both the treatment groups had signs and symptoms of nephrotoxicity and hepatotoxicity.

## Discussion

At present, VL remains one of the most neglected diseases globally [2]. To eliminate this endemic disease by 2015, a Tripartite Memorandum of Understanding agreement was signed in 2005 by the Governments of India, Nepal and Bangladesh wherein MF monotherapy was introduced as a first-line treatment [19]. However, of late, efficacy of MF has been declining steadily (96.7% to 72%) and its teratogenic potential remains a major concern in these areas where women are from low income groups and direct counseling is difficult, which limits its use in settings where the directly observed treatment is possible [10], [11], [20]. Other long-term treatment options, namely AmB (15 i.v injections over 30 days), pose a remarkable burden on the patient as well as health infrastructure [13]. Thus, short-course effective treatment regimens are greatly needed for the treatment of VL.

In this study, in the mITT population, efficacy of single day infusions of ABLE 15 mg/kg/day was satisfactory with an initial cure rate of 95.9% compared with 100% for LAmB. The difference in the initial cure rate was statistically significant between the groups (p = 0.028). However, this should be interpreted with caution, as in field settings LAmB is used as a single bolus dose of 10 mg/kg/day, compared to 15 mg/kg/day as was used in this study. This might have caused differences in the intended exposure to the treatment drug between groups. These results are in line with the results observed in a previous Phase II ABLE study [17].

Weight gain and decrease in spleen size were similar in both groups. The ABLE group showed greater increase in Hb concentration (79.4%) compared to the LAmB group (65.6%). Overall, clinical improvement (Day 30) was comparable (p = 0.6414) between the ABLE (98.9%) and LAmB groups (98.4%). Furthermore, the proportion of patients with no symptoms of relapse or showing no clinical signs of disease (definitive cure) was 85.9% in the ABLE group and 98.4% in the LAmB group.

The safety and tolerability of ABLE observed over 6 months duration was consistent with the earlier Phase II ABLE studies [17], [18]. Infusion related pyrexia and chills were the most common drug-related adverse events in both groups. These were mainly attributed to non-administration of premedication. However, in the field and in most studies, the patients are given premedication to prevent infusion related reactions [21]. No patient showed signs and symptoms of nephrotoxicity and hepatotoxicity, which was consistent with results from previous Phase II studies [17], [18]. Thus, the efficacy and safety results indicate that the treatment with ABLE is efficacious and safe.

Apart from efficacy and safety, which are important aspects of any drug or formulation, secondary aspects such as cost-effectiveness, affordability, better compliance and ease of availability must also be considered to assess the feasibility of its use. The necessity to assess secondary aspects of drug become even more important as VL mainly affects poor and neglected populations in East Africa and the Indian sub-continent [22]. It is estimated that illness due to VL may result in loss of income for up to 60% of the total household cost [23]. Thus, single-dose treatment regimens will not only reduce the hospital cost, but also drastically reduce the economic burden on the family [15]. Additionally, it has been proposed that extensive use of AmB and its formulations for treatment of VL may aid in decreasing the incidence of post-kala-azar dermal leishmaniasis, a potential reservoir of disease [24]. However, it is necessary to closely monitor and counsel patients about infrequent cases of post-kala-azar dermal leishmaniasis that tend to occur post-treatment with novel AmB formulations [25], [26].

In addition to poor economic conditions, co-infection with human immunodeficiency virus has changed the classical picture of VL in India, particularly in Bihar [27], [28]. Co-infected patients generally have poor response to the treatment, which leads to frequent relapses and high mortality [29]. In this context, it is imperative that such patients are diagnosed and treated appropriately through active case detection approaches as co-infected patients tend to transmit more virulent strains of VL.

In summary, novel formulations of AmB due to its high therapeutic index, short treatment courses and favorable safety profile have become an attractive treatment option [20]. However, in India, which bears the highest burden of VL, LAmB is not yet registered and is imported under special license, which can be a limiting factor in its availability. Also, India is not a recipient of the LAmB donation initiative, which may further impact its availability to poorer patients [16]. In addition, in India, LAmB is available at a preferential price only in limited quantities through specific treatment channels, thereby limiting its access to several patients infected with the disease. On the other hand, the ABLE formulation has been registered in India and can be supplied in liberal quantities, locally. Thus, favorable efficacy and safety profile, treatment compliance, affordability and availability of ABLE formulation make it a strong candidate for treatment of VL and for inclusion into the VL elimination program.

### Conclusions

In this study, ABLE 15 mg/kg single bolus was found to be efficacious, safe and well tolerated in patients with VL. In addition, its ancillary properties such as favorable applicability and compliance (due to single dose administration), low cost and unrestricted supply, make it a suitable option for VL treatment in endemic countries.

## Supporting Information

Supporting Information S1 **CONSORT checklist.**
(DOC)

Supporting Information S2 **Protocol.**
(DOCX)

## References

[1] Sinha PK, Roddy P, Palma PP, Kociejowski A, Lima MA, et al (2010) Effectiveness and safety of liposomal amphotericin B for visceral leishmaniasis under routine program conditions in Bihar, India. Am J Trop Med Hyg 83: 357–364. 20682882 10.4269/ajtmh.2010.10-0156 PMC2911185

[2] Leishmaniasis: Epidemiology and access to medicines. Available: http://www.who.int/leishmaniasis/resources/Leishmaniasis_worldwide_epidemiological_and_drug_access_update.pdf. Accessed 30 July 2013.

[3] Perry D, Dixon K, Garlapati R, Gendernalik A, Poche D, et al (2013) Visceral leishmaniasis prevalence and associated risk factors in the saran district of Bihar, India, from 2009 to July of 2011. Am J Trop Med Hyg 88: 778–784. 23382167 10.4269/ajtmh.12-0442 PMC3617869

[4] van Griensven J, Balasegaram M, Meheus F, Alvar J, Lynen L, et al (2010) Combination therapy for visceral leishmaniasis. Lancet Infect Dis 10: 184–194. 20185097 10.1016/S1473-3099(10)70011-6

[5] Sundar S (2001) Drug resistance in Indian visceral leishmaniasis. Trop Med Int Health 6: 849–854. 11703838 10.1046/j.1365-3156.2001.00778.x

[6] Bhattacharya SK, Sinha PK, Sundar S, Thakur CP, Jha TK, et al (2007) Phase 4 trial of miltefosine for the treatment of Indian visceral leishmaniasis. J Infect Dis 196: 591–598. 17624846 10.1086/519690

[7] Jha TK, Sundar S, Thakur CP, Bachmann P, Karbwang J, et al (1999) Miltefosine, an oral agent, for the treatment of Indian visceral leishmaniasis. N Engl J Med 341: 1795–1800. 10588964 10.1056/NEJM199912093412403

[8] Rahman M, Ahmed BN, Faiz MA, Chowdhury MZ, Islam QT, et al (2011) Phase IV trial of miltefosine in adults and children for treatment of visceral leishmaniasis (kala-azar) in Bangladesh. Am J Trop Med Hyg 85: 66–69. 21734127 10.4269/ajtmh.2011.10-0661 PMC3122346

[9] Sundar S, Jha TK, Thakur CP, Engel J, Sindermann H, et al (2002) Oral miltefosine for Indian visceral leishmaniasis. N Engl J Med 347: 1739–1746. 12456849 10.1056/NEJMoa021556

[10] Sundar S, Olliaro PL (2007) Miltefosine in the treatment of leishmaniasis: Clinical evidence for informed clinical risk management. Ther Clin Risk Manag 3: 733–740. 18472998 PMC2376078

[11] Sundar S, Singh A, Rai M, Prajapati VK, Singh AK, et al (2012) Efficacy of miltefosine in the treatment of visceral leishmaniasis in India after a decade of use. Clin Infect Dis 55: 543–550. 22573856 10.1093/cid/cis474

[12] Sinha PK, Jha TK, Thakur CP, Nath D, Mukherjee S, et al (2011) Phase 4 pharmacovigilance trial of paromomycin injection for the treatment of visceral leishmaniasis in India. J Trop Med 2011: 645203. 22174722 10.1155/2011/645203 PMC3235903

[13] Wasan KM, Wasan EK, Gershkovich P, Zhu X, Tidwell RR, et al (2009) Highly effective oral amphotericin B formulation against murine visceral leishmaniasis. J Infect Dis 200: 357–360. 19545212 10.1086/600105

[14] Sundar S, Agrawal G, Rai M, Makharia MK, Murray HW (2001) Treatment of Indian visceral leishmaniasis with single or daily infusions of low dose liposomal amphotericin B: randomised trial. BMJ 323: 419–422. 11520836 10.1136/bmj.323.7310.419 PMC37549

[15] Sundar S, Jha TK, Thakur CP, Mishra M, Singh VP, et al (2003) Single-dose liposomal amphotericin B in the treatment of visceral leishmaniasis in India: a multicenter study. Clin Infect Dis 37: 800–804. 12955641 10.1086/377542

[16] Balasegaram M, Ritmeijer K, Lima MA, Burza S, Ortiz Genovese G, et al (2012) Liposomal amphotericin B as a treatment for human leishmaniasis. Expert Opin Emerg Drugs 17: 493–510. 23167833 10.1517/14728214.2012.748036 PMC3518293

[17] Sundar S, Singh A, Agarwal D, Rai M, Agrawal N, et al (2009) Safety and efficacy of high-dose infusions of a preformed amphotericin B fat emulsion for treatment of Indian visceral leishmaniasis. Am J Trop Med Hyg 80: 700–703. 19407109

[18] Sundar S, Chakravarty J, Agarwal D, Shah A, Agrawal N, et al (2008) Safety of a pre-formulated amphotericin B lipid emulsion for the treatment of Indian Kala-azar. Trop Med Int Health 13: 1208–1212. 18664241 10.1111/j.1365-3156.2008.02128.x

[19] Stauch A, Duerr HP, Dujardin JC, Vanaerschot M, Sundar S, et al (2012) Treatment of visceral leishmaniasis: model-based analyses on the spread of antimony-resistant L. donovani in Bihar, India. PLoS Negl Trop Dis 6: e1973. 23285309 10.1371/journal.pntd.0001973 PMC3527335

[20] den Boer ML, Alvar J, Davidson RN, Ritmeijer K, Balasegaram M (2009) Developments in the treatment of visceral leishmaniasis. Expert Opin Emerg Drugs 14: 395–410. 19708817 10.1517/14728210903153862

[21] Sundar S, Mehta H, Suresh AV, Singh SP, Rai M, et al (2004) Amphotericin B treatment for Indian visceral leishmaniasis: conventional versus lipid formulations. Clin Infect Dis 38: 377–383. 14727208 10.1086/380971

[22] Chappuis F, Sundar S, Hailu A, Ghalib H, Rijal S, et al (2007) Visceral leishmaniasis: what are the needs for diagnosis, treatment and control? Nat Rev Microbiol 5: 873–882. 17938629 10.1038/nrmicro1748

[23] Boelaert M, Meheus F, Robays J, Lutumba P (2010) Socio-economic aspects of neglected diseases: sleeping sickness and visceral leishmaniasis. Ann Trop Med Parasitol 104: 535–542. 21092391 10.1179/136485910X12786389891641

[24] Thakur CP, Kumar A, Mitra G, Thakur S, Sinha PK, et al (2008) Impact of amphotericin-B in the treatment of kala-azar on the incidence of PKDL in Bihar, India. Indian J Med Res 128: 38–44. 18820357

[25] Das VN, Ranjan A, Pandey K, Singh D, Verma N, et al (2012) Clinical epidemiologic profile of a cohort of post-kala-azar dermal leishmaniasis patients in Bihar, India. Am J Trop Med Hyg 86: 959–961. 22665600 10.4269/ajtmh.2012.11-0467 PMC3366539

[26] Burza S, Sinha PK, Mahajan R, Sanz MG, Lima MA, et al (2014) Post Kala-Azar dermal leishmaniasis following treatment with 20 mg/kg liposomal amphotericin B (Ambisome) for primary visceral leishmaniasis in Bihar, India. PLoS Negl Trop Dis 8: e2611. 24392171 10.1371/journal.pntd.0002611 PMC3879248

[27] World Health Organization Control of the leishmaniases: Report of a meeting of the WHO Expert Committee on the Control of Leishmaniases, Geneva, 22–26 March 2010. WHO technical report series; no. 949. Geneva: WHO.

[28] Mathur P, Samantaray JC, Vajpayee M, Samanta P (2006) Visceral leishmaniasis/human immunodeficiency virus co-infection in India: The focus of two epidemics. J Med Microbiol 55: 919–922. 16772420 10.1099/jmm.0.46574-0

[29] Cota GF, de Sousa MR, Fereguetti TO, Rabello A (2013) Efficacy of anti-leishmania therapy in visceral leishmaniasis among HIV infected patients: A systematic review with indirect comparison. PLoS Negl Trop Dis 7: e2195. 23658850 10.1371/journal.pntd.0002195 PMC3642227

