# Supplementary material for: Efficacy and Safety of Amphotericin B Emulsion versus Liposomal Formulation in Indian Patients with Visceral Leishmaniasis: A Randomized, Open-Label Study
Source: PLoS Negl Trop Dis. 2014 Sep 18;8(9):e3169. doi: 10.1371/journal.pntd.0003169 (PMC4169371; doi:10.1371/journal.pntd.0003169)
Supplement: Supporting Information S2 — Protocol. (DOCX) [file pntd.0003169.s002.docx]

**Controlled Copy No:**

**Study BSV-AMBE III-KA-0908/ Protocol Version 3.0 date 23 Jun 09/2026**

**Printed By: Benimadhavsih Rajput**

**Printed Date: 23-Jun-2009**

A Prospective, Multicentric, Randomized, Two Arm, Open‑Label Phase III Study To Assess Efficacy And Safety Of Infusion Of Amphomul^®^ (Amphotericin B Emulsion) As Compared To AmBisome In Patients Of Visceral Leishmaniasis (Kala‑Azar)

**Confidentiality Statement**

The information provided in this protocol is strictly confidential and the sole property of Bharat Serums and Vaccines Ltd. It has been released for review only by Clinical Investigators, appropriate Ethics Committees and Regulatory Agencies. No part of the protocol should ever be reproduced or transmitted; no disclosure whatsoever must take place without written authorization from Bharat Serums and Vaccines Ltd, except to the extent necessary to ensure ethical conduct of the study.

# TITLE PAGE

A Prospective, Multicentric, Randomized, Two Arm, Open-label Phase III Study to Assess Efficacy and Safety of Infusion of Amphomul^®^ (Amphotericin B Emulsion) as Compared to AmBisome in Patients of Visceral Leishmaniasis (Kala‑azar)

**Study code:** BSV-AMBE III-KA-0908

**Version no.:** 3.0

**Version date:**  23‑Jun‑09

**Investigational product:** Amphomul^®^ *(*Amphotericin B Emulsion*)*

**Comparator product:** AmBisome

Contract Research Organization (CRO)

**SIRO Clinpharm Pvt. Ltd**.

DIL Premises, 2^nd^ Floor

Swami Vivekanand Road,

Near Tatwagyan Vidyapeeth,

Ghodbunder Road, Thane West ‑ 400607, India

Tel: +91 22 2584 8000

Fax: +91 22 2584 8275-76

SPONSOR

**Bharat Serums and Vaccines Ltd.**

17^th^ Floor, Hoechst House

Nariman Point

Mumbai - 400 021, INDIA

Tel: +91 22 6656 0900

Fax: +91 22 6752 2699

#

# VERSION CONTROL PAGE

- - This is the Version 3.0 of the protocol dated 23‑Jun‑09. This protocol is preceded by the Version 2.0 of the Protocol dated: 18‑Dec‑08. This protocol is meant for regulatory and ethics committee submissions and approvals.
  - The changes to version 2.0 are summarized as follows:

| **S. No.** | **Original Text** | **Change Text** | **Reason for change** | **Requested by** |
| --- | --- | --- | --- | --- |
|  | In section 18.2.3 (Adverse Events), the text under the title “Follow‑up of AEs was-  If any AE is present when a patient completes the study or if a patient is withdrawn from the study, the patient will be re-evaluated within 1 week | The text has been changed to -  If any AE is present when a patient completes the study or if a patient is withdrawn from the study, the patient will be re-evaluated within 2 weeks | As the follow-up visit within a short period of 1week of the study completion/withdrawal will cause inconvenience to the patient and no significant change is expected in the event during this short follow-up period, hence the patient can be re-evaluated in 2 weeks. | Medical Monitor |
|  | In section 8 (Study summary), the inclusion criteria was-  hemoglobin≥5mg/dL | The text of the inclusion criteria has been changed to-  hemoglobin≥5g/dL  Similar change was also done in Section 12.3 | Conventional unit of hemoglobein is g/dL | Medical Monitor |
|  | In section 8 (Study summary), the text under the Visits and assessments done for Day 30 was -  Laboratory investigations done:-Hematology, Renal and Liver function tests, electrolytes as outlined in the screening section at investigator’s discretion | The text has been changed to -  Laboratory investigations done:-Hematology, Renal and Liver function tests, electrolytes as outlined in the screening section | At visit 2, Day 30, the mentioned laboratory investigations form important part of efficacy analysis and hence are to be performed but at Day 45, they can be performed at investigator’s discretion | Medical Monitor |
|  | In section 18.4 (Adverse Events), the text under the title Reporting of SAEs was –  All SAEs should be reported within 24 hours of Investigator’s knowledge of the event to SIRO and the IRB/IEC. Confirmation of submission to the IRB/IEC must be forwarded to SIRO. | The text is changed to -  All SAEs should be reported within 24 hours to SIRO and within 7 calendar days to the IRB/IEC on Investigator’s knowledge of the event. Confirmation of submission to the IRB/IEC must be forwarded to SIRO. | This change is suggested to have clarity on the difference in the reporting timeline by the Investigator to SIRO and IRB/IEC. All the SAE’s are to be reported to IRB/IEC within 7 calendar days, this is as per SIRO Safety SOP. | Medical Monitor |
|  | In section 15 (Study formulation) the text under the sub-section 15.2.1‑title Drug storage was –  They must be kept in refrigerator (+2°C to +8°C or 36°F to 46°F) and must not be frozen | The text is changed to -  They must be stored below +25°C and must not be frozen  Similar change was also done in Section 15.5 | From the stability data available from the sponsor, the investigational product can be stored below +25°C considering the demographic factors of the sites. Also, at some sites there might be inadequacy of storage between 2-8°C. | Project Manager |

#

# LIST OF ABBREVIATIONS

| AE | Adverse Event |
| --- | --- |
| ALT | Alanine aminotransferase |
| AMB | Amphotericin B |
| ANC | Absolute Neutrophil Count |
| AST | Aspartate aminotransferase |
| BUN | Blood Urea Nitrogen |
| CRF | Case Report Form |
| CRO | Contract Research Organization |
| CTC | Common Terminology Criteria |
| DCGI | Drugs Controller General, India |
| DLT | Dose Limiting Toxicity |
| ECG | Electrocardiogram |
| EOS | End of Study |
| ESR | Erythrocyte Sedimentation Rate |
| GCP | Good Clinical Practice |
| GMP | Good Manufacturing Practices |
| HBsAg | Hepatitis B surface antigen |
| HCV | Hepatitis C virus |
| HIV | Human Immunodeficiency Virus |
| IB | Investigator’s Brochure |
| ICH | International Conference on Harmonization f Technical Requirements for Registration of Pharmaceuticals for Human Use |
| ICMR | Indian Council of Medical Research |
| IEC | Independent Ethics Committee |
| IP | Investigational Product |
| IRB | Institutional Review Board |
| IRT | Infusion Related Toxicity |
| ITT | Intent To Treat |
| IV | Intravenous |
| LAR | Legally Acceptable Representative |
| MedDRA | Medical Dictionary for Regulatory Activities |
| MITT | Modified Intent To Treat |
| NCI | National Cancer Institute |
| PI | Principal Investigator |
| PP | Per Protocol |
| RBC | Red Blood Corpuscles |
| rK39 | Recombinant K39 Protein |
| SAE | Serious Adverse Event |
| SOP | Standard Operating Procedure |
| ULN | Upper Limit of Normal |
| UPT | Urine Pregnancy Test |
| WBC | White Blood Cells |

# TABLE OF CONTENTS

[1. TITLE PAGE 2](#_Toc217795557)

[2. VERSION CONTROL PAGE 3](#_Toc217795558)

[3. LIST OF ABBREVIATIONS 3](#_Toc217795559)

[4. TABLE OF CONTENTS 3](#_Toc217795560)

[5. RESPONSIBLE PERSONNEL 3](#_Toc217795561)

[6. SIGNATURE PAGE 3](#_Toc217795562)

[7. INVESTIGATOR ENDORSEMENT 3](#_Toc217795563)

[8. STUDY SUMMARY 3](#_Toc217795564)

[9. INTRODUCTION 3](#_Toc217795565)

[9.1 Disease Scenario in India (Visceral Leishmaniasis) 3](#_Toc217795566)

[9.2 Proposed Therapeutic Indication 3](#_Toc217795567)

[9.3 Description of Investigational product (Amphomul^®^) 3](#_Toc217795568)

[9.4 Summary of Preclinical Studies with Amphomul^®^ 3](#_Toc217795569)

[9.5 Clinical Studies 3](#_Toc217795570)

[9.6 Rationale for the study 3](#_Toc217795571)

[10. STUDY OBJECTIVES and ENDPOINTS 3](#_Toc217795572)

[10.1 OBJECTIVES 3](#_Toc217795573)

[10.1.1 Primary 3](#_Toc217795574)

[10.1.2 Secondary 3](#_Toc217795575)

[10.2 ENDPOINTS 3](#_Toc217795576)

[10.2.1 Primary 3](#_Toc217795577)

[10.2.2 Secondary 3](#_Toc217795578)

[11. STUDY DESCRIPTION (INVESTIGATIONAL PLAN) 3](#_Toc217795579)

[11.1 Basic Principles and Ethical Considerations 3](#_Toc217795580)

[11.2 Sample Size 3](#_Toc217795581)

[11.3 Study Design 3](#_Toc217795582)

[11.4 Flow of events for the study 3](#_Toc217795583)

[11.5 Study Duration, Treatment Interruption and Termination 3](#_Toc217795585)

[12. STUDY POPULATON 3](#_Toc217795586)

[12.1 Sample size 3](#_Toc217795587)

[12.2 Target Population 3](#_Toc217795588)

[12.3 Inclusion Criteria 3](#_Toc217795589)

[12.4 Exclusion Criteria 3](#_Toc217795590)

[12.5 Patient Information and Informed Consent 3](#_Toc217795591)

[12.6 Patient Completion and Withdrawal 3](#_Toc217795592)

[12.6.1 For each patient, according to the protocol, the study is deemed 3](#_Toc217795593)

[12.6.2 Procedures for handling patients who discontinue the study prematurely 3](#_Toc217795594)

[12.6.3 Recording Patient Withdrawals 3](#_Toc217795595)

[12.6.4 Replacement of Patients Withdrawn 3](#_Toc217795596)

[13. COURSE OF STUDY 3](#_Toc217795597)

[15. STUDY CALENDAR 3](#_Toc217795598)

[15. STUDY FORMULATION 3](#_Toc217795599)

[15.1 Product information 3](#_Toc217795600)

[15.1.1 Investigational product (Amphomul^®^) 3](#_Toc217795601)

[15.1.2 Reference product (AmBisome) 3](#_Toc217795602)

[15.2 Supply, storage and accountability of study drug 3](#_Toc217795603)

[15.2.1 Drug storage 3](#_Toc217795604)

[15.2.2 Drug supplies 3](#_Toc217795605)

[15.2.2 Drug accountability 3](#_Toc217795606)

[15.3 Randomization and Blinding 3](#_Toc217795607)

[15.4 Code Handling and Breaking 3](#_Toc217795608)

[15.5 Packaging and Labeling of Study drug 3](#_Toc217795609)

[15.6 Dosing schedule and Administration 3](#_Toc217795610)

[15.6.1 Investigational product (Amphomul^®^) 3](#_Toc217795611)

[15.6.2 Reference product (AmBisome) 3](#_Toc217795612)

[15.6.3 Premedication 3](#_Toc217795613)

[15.6.4 Duration and Interruption of study drug treatment 3](#_Toc217795614)

[15.7 Medication Compliance 3](#_Toc217795615)

[16. CONCURRENT THERAPY 3](#_Toc217795616)

[16.1 Concurrent Medication 3](#_Toc217795617)

[16.1.1 Prohibited Medication 3](#_Toc217795618)

[16.1.2 Permitted Medications 3](#_Toc217795619)

[16.2 Rescue Medication 3](#_Toc217795620)

[17. ASSESSMENT OF EFFICACY 3](#_Toc217795621)

[17.1 Efficacy endpoints 3](#_Toc217795622)

[17.2 Efficacy evaluation 3](#_Toc217795623)

[18. ASSESSMENT OF SAFETY 3](#_Toc217795624)

[18.1 Definitions 3](#_Toc217795625)

[18.1.1 Adverse Events 3](#_Toc217795626)

[18.1.2 Adverse Drug Reactions 3](#_Toc217795627)

[18.1.3 Serious Adverse Events 3](#_Toc217795628)

[18.1.4 Unexpected/ Unlisted Adverse Event 3](#_Toc217795629)

[18.1.5 Expedited Safety Report 3](#_Toc217795630)

[18.2 Adverse Event Collection Period and Follow up 3](#_Toc217795631)

[18.2.1 Start of AE collection period 3](#_Toc217795632)

[18.2.2 End of AE collection period 3](#_Toc217795633)

[18.2.3 Follow Up of Adverse Events 3](#_Toc217795634)

[18.3 Recording of Adverse Events 3](#_Toc217795635)

[18.3.1 General 3](#_Toc217795636)

[18.3.2 Assessment of intensity 3](#_Toc217795637)

[18.3.3 Assessment of Causality 3](#_Toc217795638)

[18.3.4 Assessment of outcome 3](#_Toc217795639)

[18.3.5 Handling of Unresolved AEs 3](#_Toc217795640)

[18.4 Reporting of SAEs 3](#_Toc217795641)

[18.5 Expedited Safety Reporting and Periodic Safety Update Reports 3](#_Toc217795642)

[18.6 Protocol Specific safety issues 3](#_Toc217795643)

[18.7 Exposure *in utero* 3](#_Toc217795644)

[19. BLOOD/TISSUE SAMPLE HANDLING AND ANALYSIS 3](#_Toc217795645)

[20. STATISTICAL ANALYSIS PLAN 3](#_Toc217795646)

[20.1 Analysis Sets 3](#_Toc217795647)

[20.2 Statistical Analysis 3](#_Toc217795648)

[20.2.1 Demographic Data 3](#_Toc217795649)

[20.2.2 Efficacy Analysis 3](#_Toc217795650)

[20.2.3 Safety Analysis 3](#_Toc217795651)

[20.2.4 Statistical Analysis of Data 3](#_Toc217795652)

[21. PROTOCOL MODIFICATIONS, DEVIATIONS and AMENDMENTS 3](#_Toc217795653)

[21.1 Deviations for a particular patient 3](#_Toc217795654)

[21.2 Amendments, if any for the study 3](#_Toc217795655)

[21.2.1 Administrative modifications 3](#_Toc217795656)

[21.2.2 Clinical modifications 3](#_Toc217795657)

[22. ADMINISTRATIVE MATTERS- ADHERENCE TO GOOD CLINICAL PRACTICE (GCP) GUIDELINES 3](#_Toc217795658)

[22.1 Regulatory requirements 3](#_Toc217795659)

[22.2 Ethics Committee 3](#_Toc217795660)

[22.3 Investigator Qualification 3](#_Toc217795661)

[22.4 Clinical Supplies and Storage 3](#_Toc217795662)

[22.5 Data Collection, Documentation and Archiving of Study Documents 3](#_Toc217795663)

[22.6 Study Initiation 3](#_Toc217795664)

[22.7 Study Monitoring and Source Data Verification 3](#_Toc217795665)

[22.8 Record Retention 3](#_Toc217795666)

[22.9 Insurance and Indemnity 3](#_Toc217795667)

[22.10 Audits and Inspection 3](#_Toc217795668)

[22.11 Data Management and Archival 3](#_Toc217795669)

[22.12 Patient Confidentiality and Data Protection 3](#_Toc217795670)

[22.13 Final Study Report and Publication 3](#_Toc217795671)

[23. REFERENCES 3](#_Toc217795672)

**Appendices**

**Appendix A:** Declaration of Helsinki

**Appendix B:** Patient Information Sheet and Informed Consent Form

**Appendix C:** Assent Form

**Appendix D:** NCI-CTC Criteria Version 3

#

# RESPONSIBLE PERSONNEL

**CRO’s Responsible Personnel**

| **Project Manager** | **Mr. Kunal Sehra**  DIL Premises, 2nd Floor  Swami Vivekanand Road,  Near Tatwagyan Vidyapeeth,  Ghodbunder Road, Thane West ‑ 400607, India  Tel: +91 22 2584 8451  Fax: +91 22 2584 8275-76  Email: kunal.sehra@siroclinpharm.com |
| --- | --- |
| **Medical and Safety Monitor** | **Dr. Swati Sharma**  DIL Premises, Ground Floor  Swami Vivekanand Road,  Near Tatwagyan Vidyapeeth,  Ghodbunder Road, Thane West ‑ 400607, India  Tel: +91 22 2584 8429  Fax: +91 22 2584 8283  Email: swati.sharma@siroclinpharm.com |
| **Project Statistician** | **Ms. Harshada Tamhankar**  DIL Premises, Ground Floor  Swami Vivekanand Road,  Near Tatwagyan Vidyapeeth,  Ghodbunder Road, Thane West ‑ 400607, India  Tel: +91 22 2584 2277  Fax: +91 22 2584 8412  Email: harshada.tamhankar@siroclinpharm.com |

**Sponsor’s Responsible Personnel**

**Name and address of person responsible for packaging and labeling of study drug**

Mr. D. G. Ranjane

Bharat Serums and Vaccines Ltd

Plot K-27. Additional MIDC,

Ambernath (E) ‑ 421 501

Tel.: +91-251‑2621081 / 83

Fax: +91-251‑2621089

E-mail: [ranjane.d@bharatserums.com](mailto:ranjane.d@bharatserums.com)

**Name and address of the Principal Investigator conducting the study**

| **Principal Investigator(s)** | **Address and contact details** | **Study Site** |
| --- | --- | --- |
| **Dr. Shyam Sundar** | Professor of Medicine,  Institute of Medical Sciences Banaras Hindu University,  Varanasi – 221 005, India  Tel: 91-542-2309493 Email:drshyamsundar@hotmail.com | Kala-azar Medical Research Center,  Rambag road,  Muzaffarpur,  Bihar- 842001 |
| **Dr. T.K. Jha** | Kalazar Research Center,  Brahmpura, Muzaffarpur,  Bihar- 842 003, India  Tel: 91 94312 38088 / 93349 14050  Email: dr_tkjha@hotmail.com | Kalazar Research Center,  Brahmpura,  Muzaffarpur,  Bihar- 842 003, India |
| **Dr. Krishna Pandey** | Rajendra Memorial Research Institute of Medical Sciences (RMRI) Agamkuan, Patna – 800 007  Tel: 91-612-2631565  Email:drkrishnapandey@yahoo.com | Rajendra Memorial Research Institute (RMRI),  Agamkuan,  Patna – 800007 |
| **Dr. C.P. Thakur** | Balaji Utthan Sansthan,  Kala Azar Research Centre,  Uma Complex  Fraser Road,  Patna - 800 001, India  Tel: 91-98352 39319  Email: cpthakur1@rediffmail.com  thakurcp@gmail.com | Balaji Utthan Sansthan, Kala Azar Research Centre, Uma Complex  Fraser Road,  Patna - 800 001 |

# SIGNATURE PAGE

A Prospective, Multicentric, Randomized, Two Arm, Open-label, Phase III Study to Assess Efficacy and Safety of Infusion of Amphomul^®^ (Amphotericin B Emulsion) as Compared to AmBisome in Patients of Visceral Leishmaniasis (Kala‑azar)

**BSV-AMBE III-KA-0908 Protocol Version 3.0 dated 23‑Jun‑2009**

**On behalf of the Sponsor – Bharat Serums and Vaccines Ltd.**

**Principal Coordinator**

Name: **Dr. Gautam V. Daftary**

Signature with date:

**On behalf of the CRO ‑ SIRO Clinpharm Pvt. Ltd.**

**Project Manager**

Name: **Mr. Kunal Sehra**

Signature with date:

**Medical and Safety Monitor**

Name:  **Dr. Swati Sharma**

Signature with date:

**Biostatistician**

Name: **Ms Harshada Tamhankar**

Signature with date:

# INVESTIGATOR ENDORSEMENT

I understand that this protocol contains information that is confidential and proprietary to Bharat Serums and Vaccines Ltd.

I have read the foregoing protocol entitled “A Prospective, Multicentric, Randomized, Two Arm, Open-label Phase III Study to Assess Efficacy and Safety of Infusion of Amphomul^®^ (Amphotericin B Emulsion) as Compared to AmBisome in Patients of Visceral Leishmaniasis (Kala‑azar)” BSV‑AMBE III‑KA-0908 Protocol Version 3.0, dated 23‑Jun‑09 and agree that it contains all necessary details for carrying out the study as described. I will conduct this protocol as outlined herein and will make a reasonable effort to complete the study within the time designated.

I will discuss the contents of this protocol to all those authorized study staff, who will assist me in conducting this trial in order to ensure that they are fully informed about the investigational product (IP) and the study.

I will also provide protocol information to my Institutional Review Board (IRB*)/*Independent Ethics Committee (IEC*)* or responsible federal authority patient to the following condition: the contents of this protocol will not be used in any other clinical trial and may not be disclosed to any other person or entity without the prior written permission of Bharat Serums and Vaccines Ltd.

Any supplemental information that may be added to this document is also confidential and proprietary to Bharat Serums and Vaccines Ltd. and must be kept in confidence in the same manner as the contents of this protocol.

| **Investigator’s Details** |  |
| --- | --- |
| Investigator’s Name | _____________________________________________ |
| Institution Name | _____________________________________________ |
| Address | _____________________________________________ |
| Phone Number(s) | _____________________________________________ |
| Fax number(s) | _____________________________________________ |
| Signature with date | ____________________________________________ |

#

# STUDY SUMMARY

**Name of Sponsor:** Bharat Serums and Vaccines Ltd.

**Investigator product:** Amphomul^®^ *(*Amphotericin B Emulsion)

**Comparator Product:** AmBisome

Title of Study: A Prospective, Multicentric, Randomized, Two Arm, Open‑label Phase III study to Assess Efficacy and Safety of Infusion of Amphomul^®^ (Amphotericin B Emulsion) as Compared to AmBisome in Patients of Visceral Leishmaniasis (Kala‑azar)

**Study Code:** BSV‑AMBE III‑KA‑0908

**Development Phase:** III

**Indication:** Visceral Leishmaniasis (Kala-azar)

**Study Design:** A Prospective, Multicenteric, Randomized (3:1), Two‑arm, Open‑label Phase III study

**Study Objectives:**

***Primary Objective***: To compare and evaluate efficacy of Amphotericin B emulsion (15 mg/kg/day as a single dose infusion) compared to AmBisome (15 mg/kg as a single dose infusion) in the treatment of Visceral Leishmaniasis (Kala-azar)

***Secondary Objective***: To assess the safety of Amphotericin B emulsion (15 mg/kg as a single dose infusion) compared to AmBisome (15 mg/kg as a single dose infusion) in the treatment of Visceral Leishmaniasis (Kala-azar).

**Patient Population:** Patients with Visceral Leishmaniasis (Kala-azar)

**Number of Centers**: 4 centers

**Total Patients:** 500

Considering a total drop out rate after randomization and major protocol deviations close to 20%, 500 patients (i.e. 375 patients in Amphomul^®^ group and 125 patients in AmBisome group) are required to be enrolled in the study to give analyzable data of at least 400 subjects.

**Study Treatment:**

- 1. Investigational drug - Amphomul^®^ (Amphotericin B Emulsion manufactured by Bharat Serums and Vaccines Ltd.), and
  2. Comparator drug - AmBisome (manufactured by Gilead Sciences, Inc.)

**Dosage and route of administration:** On the basis of Phase II study results (BSV‑AMBE II‑KA-0706), dose of Amphomul^®^ was decided for this Phase III study. Patients will be randomized to receive either infusion of Amphomul^®^ 15 mg/kg as a single dose administered over 4 to 6 hours or AmBisome 15 mg/kg administered as a single dose infusion over 4 to 6 hours.

**Treatment strategy:**

- The patients will be randomized to receive either Amphomul^®^ or AmBisome (ratio 3:1).
- **All the patients recruited will be hospitalized for 7 days counting from Day 1 of therapy irrespective of the regimen used.**
- **No premedication will be administered prior to infusion of Amphomul and AmBisome.**
- **Safety assessment will be performed at Day 7 and Day 30.**
- **Patient will be discharged from the hospital on Day 7.**

**Route of Administration:** Intravenous (IV)

**Treatment Duration:** Single day as stated above

**Assessment of Dose Limiting Toxicity (DLT)**:

- Recruitment of all subsequent patients in any dosage regimen will be stopped or discontinued if the overall percentage of patients in the study with DLT is >33 %. This criterion will only be applicable after randomization of 25^th^ patient in either arm.
- DLT of the study drugs for this study is defined as:

i) Any drug related grade III or higher AE classified as nephrotoxicity/ hepatotoxicity evaluated as per National Cancer Institute Common Technology Criteria for adverse events (AE) version 3 i.e. (NCI-CTC-AE-v3).

ii) Any other drug related grade IV or higher toxicity except hematological toxicity (evaluated as per NCI-CTC-AE-v3).

iii) If patient’s hematological parameter is in CTC grade III or grade IV at baseline, then a DLT would be considered if there is reduction of ≥33% from baseline.

iv) If patient’s hematological parameter is within normal range or is in CTC grade I or CTC grade II at baseline, then a DLT would be considered if the parameter drops to grade IV or higher.

- All the patients will be assessed for DLT up to Day 7 after the study drug administration.

**Assessment of Safety Criteria:**

- **After the splenic aspirate score assessment at Day 30 of study, patients who have score of 0 would be considered to have achieved initial cure.**
- **Patients who have score of +1 on Day 30 will be re-examined at Day 45. If the score is 0 on Day 45, these patients will also be considered to have achieved Initial Cure.**
- **Patients with score >+1 at Day 30 and/or >0 at Day 45 will be considered as treatment failures and will be discontinued from the study.**
- The safety data comprising of AEs, serious adverse events (SAEs) and laboratory data will be assessed on Day 7 and Day 30.

**Criteria for Inclusion:**

Following patients will be included in the study -

1. Male or female patients aged between 5 to 65 years (both inclusive).
2. Patient/patient’s legally acceptable representative (LAR)/impartial witness is willing and able to give written informed consent to participate in the study.
3. Clinical signs and symptoms of Visceral Leishmaniasis (fever of over 2 weeks duration and splenomegaly)
4. Presence of amastigotes (*Leishmania-Donovani* bodies) at prescreening detected by recombinant K39 protein (rK39) dipstick test with confirmation of Kala-azar by splenic or bone marrow aspirate smear examination.
5. No previous treatment for Visceral Leishmaniasis within 30 days from screening.
6. Non-pregnant, non-lactating females of age ≥5 years, and woman of childbearing potential (any woman who has reached menarche) who are willing to use acceptable methods of contraception like long term acting injections ex. Depo-Provera.
7. Negative Urine pregnancy test (UPT) in all women physiologically capable of becoming pregnant (any woman who has reached menarche)
8. Hemoglobin ≥5 g/dL
9. White blood cells (WBC) count ≥1000/cmm
10. Platelet count ≥50000/cmm
11. Alanine amino transferase (ALT), aspartate amino transferase (AST) and alkaline phosphatase ≤2.5 times the upper limit of normal (ULN)
12. Prothrombin time ≤4 seconds above the control.
13. Normal serum potassium levels
14. Negative tests for Human immunodeficiency virus (HIV), Hepatitis C virus (HCV) and Hepatitis B surface antigen (HBsAg).

**Criteria for Exclusion:**

Patients who meet any of the following criteria at screening or prior to treatment assignment have to be excluded from study entry:

1. Patients with past history of treatment with Amphotericin B or any other drug for Visceral Leishmaniasis within 30 days prior to screening.
2. Patients positive for HIV, HCV and HBsAg infection, immunocompromised patients (through history).
3. A history or evidence of any concurrent disease that may be serious or life threatening, significant hematological, cardiac, hepatic, renal, respiratory, neurological or metabolic disease or any condition which, in the opinion of the investigator, may constitute a safety concern or interfere with the evaluation of the study objectives or may prevent the patient from the completing study therapy or subsequent follow-up may have a significant impact on the outcome of the study.
4. Concurrent diabetes, tuberculosis or bacterial pneumonia (on chest X-ray, erythrocyte sedimentation rate (ESR) at screening, past history) or any other infectious or major psychiatric disease.
5. Patients with concurrent Malaria will not be included in the trial. Parascreen antigen test for detection of malarial parasites will be performed at screening visit. Patients diagnosed with malaria could be rescreened for enrollment in the trial after adequate treatment of Malaria; and if the parascreen antigen test for detection of malarial parasites is found negative then the patient could be enrolled in the trial.
6. History of major surgery within 2 weeks prior to screening
7. Blood urea nitrogen (BUN) and serum creatinine >1.5 times ULN, and serum total bilirubin >1.5 times ULN
8. Proteinuria ≥2+
9. Pregnant or nursing women
10. History of alcoholism or illicit drug use/ abuse, or any condition associated with poor compliance
11. Known hypersensitivity to Amphotericin B, inactive ingredients of Amphomul^®^ formulation, inactive ingredients of AmBisome formulation.
12. Patients receiving any of the medications prohibited by the study protocol.
13. Simultaneous participation in another trial or received any IP <30 days prior to enrolment.
14. Any condition which in the investigator’s opinion may prevent the patient from completing the study therapy or follow-up.

Visits and Assessments Done

Screening visit Visit 0 (Day ‑7 to Day 0)

- Informed consent will be obtained.
- Detailed physical examination
- Vital signs will be recorded
- Prescreening rK39 dipstick test
- Patients will be evaluated for inclusion/exclusion criteria, demography and medical history.
- Splenic aspirate will be done only if rK39 dipstick test is positive (Prothrombin time will have to be carried out before splenic aspiration and procedure will be done only if result is ≤4 seconds above the control.)
- Hematology investigations (Hemoglobin, RBC, ESR, platelets, total and differential leukocyte count)
- Biochemistry investigations including the following:
  - - Liver function tests (Serum ALT, AST, alkaline phosphatase, total bilirubin, serum albumin, prothrombin time)
    - Renal function tests (serum creatinine, BUN*)*
    - Electrolytes (sodium, potassium, magnesium)
- HIV test, HCV and HBsAg tests.
- Fasting blood sugar test
- Parascreen antigen test for detection of malarial parasites
- X-ray chest and 12-lead electrocardiogram (ECG)
- UPT (for females of child bearing potential only)
- Urine analysis (routine, microscopy)
- Previous and concomitant medications will be recorded.

**Visit 1 (Day 1)**

- The patients will be re-evaluated clinically. If the eligibility criteria are satisfied, patient will be included in the study.
- Study drug administration
- Vital signs will be recorded
- Laboratory investigations will be done at Investigator’s discretion.
- Monitoring, recording and reporting for AEs
- Previous and concomitant medications will be recorded.
- DLT Assessment will be performed

**Visit 1 - Day 7**

- Thorough physical examination will be done and vital signs assessed.
- Safety assessment (AEs, SAEs, IRTs, check for nephrotoxicity and hepatotoxicity and laboratory values for different parameters) will be done
- Laboratory investigations done:-Hematology, Renal and Liver function tests, electrolytes as outlined in the screening section
- Vital signs will be recorded
- Monitoring, recording and reporting for AEs
- Previous and concomitant medications will be recorded.
- DLT assessment will be done for all the patients

**Visit 2 (Day 30) (+/- 5 days):**

- Thorough clinical examination will be done
- Splenic aspirate will be done (Prothrombin time will have to be carried out before splenic aspiration and procedure will be done only if result is ≤4 seconds above the control.) and parasitological cure will be graded.
- Safety assessment (AEs, SAEs; IRTs, check for nephrotoxicity and hepatotoxicity and laboratory values for different parameters) will be done
- Vital signs will be recorded
- Laboratory investigations done:-Hematology, Renal and Liver function tests, electrolytes as outlined in the screening section
- Monitoring, recording and reporting for AEs
- Previous and concomitant medications will be recorded.

**Visit 2 - Day 45 (Additional visit) (+/- 5 days):**

- This visit will be done only for those patients who did not have complete parasitological cure on Day 30 and had a splenic aspirate score of +1. If the score is +1 and above, the patients will be considered as treatment failures and will be discontinued from the study.. Patient will be given rescue medication AmBisome 5 mg/kg IV on Days 1, 3, 5 and 7 or alternative antileishmanial drug in appropriate doses as deemed fit by the Investigator.
- Thorough clinical examination will be done
- Vital signs will be recorded
- Splenic aspirate will be done (Prothrombin time will have to be carried out before splenic aspiration and procedure will be done only if result is ≤4 seconds above the control.) and parasitological cure will be graded.
- Laboratory investigations done:-Hematology, Renal and Liver function tests, electrolytes at investigator’s discretion
- Monitoring, recording and reporting for AEs
- Previous and concomitant medications will be recorded.

**Visit 3 (End of study [EOS] visit- 6 months after study drug administration) (-2 weeks to +4 weeks):**

- Thorough clinical examination will be done
- Vital signs will be recorded
- Safety assessment (AEs, SAEs, IRTs and check for nephrotoxicity and hepatotoxicity and laboratory values for different parameters) will be done
- Laboratory investigations will be done:-Hematology, Renal and Liver function tests, electrolytes as outlined in the screening section
- Splenic aspirate will be done to assess parasitological cure only in patients in whom a relapse of the illness is indicated, in others only clinical assessment will be done to define definitive cure at 6 months (Prothrombin time will have to be carried out before splenic aspiration and procedure will be done only if result is ≤4 seconds above the control)
- Monitoring, recording and reporting for AEs
- Previous and concomitant medications will be recorded.

Criteria for Efficacy and Safety Evaluation:

**Efficacy:** Patients will be assessed for initial cure on the basis of splenic aspirate score on Day 30 after first dose of study drug as follows:

| **Splenic aspirate score** | **Interpretation and action to be taken** |
| --- | --- |
| 0 | At Day 30:  Initial cure achieved; Follow-up till 6 months after study drug administration |
| +1 | Repeat confirmatory splenic aspirate at 15^th^ day after Day 30 aspirate (i.e. Day 45 of study)  At Day 45:  0 ‑ Initial cure achieved, follow-up till 6 months after study drug administration.  1+ and above - The patients will be considered as treatment failures. The patients will be withdrawn from study and given rescue medication as deemed fit by the PI. |
| Above +1 | At Day 30:  These patients will be considered as treatment failures and will be discontinued from the study and given rescue medication as deemed fit by the PI. |

**Efficacy Criteria**:

- Initial cure (splenic aspirate score 0 on Day 30 or on Day 45)
- Clinical improvement on Day 30 after study drug administration
  - Absence of fever; and >1 out of following:
    - Increase in hemoglobin concentration by >10% compared to baseline
    - Any weight gain compared to the baseline visit
    - Decrease in spleen size by >33% from baseline
- Definitive cure:

1. Initial cure at Day 30 or Day 45
2. No clinical signs and symptoms of relapse of Kala-azar for 6 months after study drug administration. If clinical signs and symptoms of relapse of the disease is suspected at any time during the 6 month follow up, splenic aspirate shall be performed at 6 months or earlier after study drug administration.

Proportion of patients achieving all three-efficacy endpoints will be compared across the two groups.

**Safety criteria:** Safety assessments will be done on Day 7 and Day 30. Safety will be assessed by the following parameters.

1. Number of AEs and SAEs
2. Incidence of IRTs
3. Incidence of nephrotoxicity and hepatotoxicity
4. Laboratory values for different parameters

All AEs, SAEs, changes in laboratory values, vital signs and physical condition will be monitored throughout the course of the study.

All tests will be performed at the local laboratory at each centre.

**Rescue medication:**

If the subject develops worsening of symptoms of Kala-azar, investigator may decide to use an alternative antileishmanial drug in appropriate doses or AmBisome 5 mg/kg IV on Days 1, 3, 5 and 7 as a rescue medication. In such situation, the subject will be discontinued from study.

**Statistical analysis:**

Statistical analysis will be performed using SAS^®^ 9.1 programming (SAS Institute Inc., USA). The evaluation of the safety and efficacy parameters of the study will be made with respect to the comparisons between the two groups under study.

# INTRODUCTION

## Disease Scenario in India (Visceral Leishmaniasis)

Kala-azar, also known as Visceral Leishmaniasis, is reported to affect 500,000 people worldwide every year. It is caused by the protozoan, *Leishmania donovani*, which is transmitted to humans through the bite of an infected female phlebotomine sand fly. Ninety percent of Visceral Leishmaniasis cases are found in Bangladesh, Brazil, India, Nepal and Sudan (*Sundar S, 2002*).

Classic Kala-azar, which is progressive and fatal if not treated, is an insidious, chronic disease that is characterized by irregular fever, anorexia, weight loss, cough, gross enlargement of the spleen and liver, mild anemia and emaciation. This may be preceded by rigors and vomiting. If untreated, Kala-azar, which is the most severe form of Leishmaniasis, has a mortality rate of nearly 100%.

Nearly 50% cases do not respond to the first line of treatment used (pentavalent antimonials) and need a second line therapy like Amphotericin B. Other drugs that have been investigated with limited success include Miltefosine. High procurement cost and possibility of treatment resistance limits the utility of this drug, hence it is not very much suitable to be used in Indian scenario.

Amphotericin B deoxycholate (Conventional Amphotericin) has also been used as a second line therapy. However, nephrotoxicity (tubular and glomerular damage), IRTs (fever, chills) and prolonged duration of treatment (nearly 20 to 30 days) are some of the factors that limit utility of this highly potent drug.

Given this scenario, reduction in duration of administration as well as toxicity caused by Amphotericin B will be of immense help improving the outcome of patients (treatment naïve or treatment resistant) who develop Visceral Leishmaniasis.

## Proposed Therapeutic Indication

Pentavalent antimonial compounds like sodium stibogluconate, meglumine antimoniate etc. have been used as a first line treatment in Kala-azar. However their use is restricted due to their toxic nature.

For resistant cases, drugs like Amphotericin B or Ketoconazole are used. Amphotericin is favored due to higher potency and cure rate achieved. However, efficacy of this drug too, is largely limited due to nephrotoxicity and IRTs.

Nephrotoxicity occurs in almost all patients receiving Conventional Amphotericin B intravenously. Both tubular and glomerular damage occurs and there is a risk of permanent impairment of renal function. IRTs (fever, chills etc.) are regularly associated with injection of Conventional Amphotericin B. Further, to achieve a ‘cure’, Conventional Amphotericin B has to be administered over 20 to 30 days. This duration of treatment poses serious difficulty for patients due to prolonged hospitalization required, as most of these patients are poor laborers who cannot afford to remain away from the work for prolonged hospitalization. Additionally, prolonged hospitalization required for treatment of Kala-azar puts an enormous burden upon the healthcare system in terms of cost as well as hours of work required.

Development of drugs, which can be administered over a short duration with minimal toxicity, is therefore urgently required. In order to reduce the toxicity of the Conventional formulation of Amphotericin B, new formulations have been developed. Amphotericin B Lipid complex (ABLC), Amphotericin B colloidal dispersion (ABCD) and liposomal Amphotericin B have proved effective experimentally and clinically. All these formulations have been shown to be effective in achieving a cure of Kala-azar at total doses of around 15 mg/kg, administered over a period of 10 days. Considering the demonstrated efficacy and relative safety of lipid formulations of Amphotericin B, it is increasingly being used as a first-line treatment in areas endemic to Kala-azar (*Sundar S, 2000*). However, their use remains limited by their high cost. It has been found that by simply mixing Amphotericin B with lipid emulsion at the time of administration allows administration of higher doses of Amphotericin B over a shorter duration and is associated with lesser toxicity. Lipid emulsions have been considered as a means of increasing tolerability of Amphotericin B (*Tabosa Do Egito, ES et al, 1996*).

## Description of Investigational product (Amphomul^®^)

Amphomul^®^ is an emulsion formulation of Amphotericin B *(*AMB*),* which is a polyene antifungal antibiotic produced from a strain of *Streptomyces nodosus*. Details of other ingredients of the formulation are mentioned in Section 15.1. Amphotericin B is designated chemically as [1R-(1R’, 3S’, 5R’, 6R’, 9R’, 11R’, 15S’, 16R’, 17R’, 18S’, 19E, 21E, 23E, 25E, 27E, 29E, 31E, 33R’, 35S’, 36R’, 37S’)] –33-[(3-amino-3, 6-dideoxy-beta-d-mannopyranosyl) oxy]–1,3,5,6,9,11,17,37-octahydroxy-15, 16, 18–trimethyl-13–oxo-14, 39–dioxabicyclo (33,3,1) nonatriaconta-19,21,23,25,27,29,31-heptaene-36–carboxylic acid. Its molecular weight is 924.09 and molecular formula is C_47_H_73_NO_17_.

**Mechanism of action:** It acts by binding to sterols in the cell membrane of susceptible fungi, with a resultant change in the permeability of the membrane. It has shown *in vitro* activity against *Asperigillus* and *Candida* with minimum inhibitory concentration generally less than 1 µg/mL. In animal studies, it has shown activity against *Asperigillus fumigatus, Candida albicans, C. guillermondil, C. stellatodae* and *C. tropicallis, Cryptococcus sp., Coccidiodomyces* sp*., Histoplasma* sp. and *Blastomyces* sp., in which the end point was clearance of microorganisms from target organs and prolonged survival of infected animals.

**Pharmacokinetics:** The pharmacokinetics of AMB after IV administration is nonlinear. Volume of distribution and blood clearance increase with increase in dose resulting in less than proportional increase in blood concentration over a dose range of 0.6 to 5.0 mg/kg/day. Extensive clinical studies on AMB suggest it to be effective against various Leishmania species in humans.

## Summary of Preclinical Studies with Amphomul^®^

A detailed description of preclinical data with Amphomul^®^ can be found in the investigator’s brochure (IB). This section will summarize the information shortly.

Pharmacokinetic profile

Male New Zealand white rabbits were tested to evaluate the pharmacokinetic parameters of the AMB formulations.

The pharmacokinetics of AMB after administration as emulsion (Amphomul^®^) is non‑linear. Amphomul^®^ is cleared from plasma at a faster rate than the conventional formulation containing sodium deoxycholate. The volume of distribution and clearance from blood is more than the Conventional formulation.

The pharmacokinetics of AMB after administration as an emulsion (Amphomul^®^) is as follows: (values in parenthesis indicate the pharmacokinetics of AMB deoxycholate)

Peak concentration (µg/mL) - 0.387±0.176 (0.576±0.006*)*

Clearance (mL/hr/kg) - 16.06±12.5 (0.54±0.28)

Apparent volume of distribution (l/kg) - 26.14 ±18.52 (1.47±1.26)

Half-life (Hours) - 6.622 ± 10.63 (1.89 ± 0.73)

AMB levels after administration as emulsion were higher in spleen and liver than as conventional formulation. AMB in emulsion formulation was rapidly distributed to tissues when injected by IV route. The ratio of drug concentration in tissues to drug concentration in blood increased disproportionately with increasing dose, suggesting that the elimination of the drug from the tissues is delayed. Peak blood levels of AMB were lower after administration of AMB emulsion than conventional formulation containing sodium deoxycholate. However, tissue levels of AMB emulsion were found to be higher than the conventional formulation.

Drug interactions

AMB is known to interact with the following drugs, which should be thus administered with caution:

Antineoplastic agents: Concurrent use may enhance potential for renal toxicity.

Corticosteroids: May potentiate hypokalemia.

Cyclosporine: Additive nephrotoxicity.

Digoxin: Nephrotoxicity may decrease digoxin clearance.

Leukocyte transfusions: acute pulmonary toxicity if given concurrently.

Skeletal muscle relaxants: Hypokalemia may potentiate skeletal muscle relaxant activity.

Zidovudine: Increased myelosuppression.

Non-steroidal anti-inflammatory drugs, sulfas, aminoglycosides, and IV contrast dyes in patients with renal insufficiency, except for dehydration due to angiotensin antagonists are well known for their nephrotoxic potential. Thus, antineoplastic drugs, corticosteroids, cyclosporin A, antifungal imidazoles if required could be administered with a close monitoring of the patients.

**Toxicology:**

Following *in vivo* studies were conducted to evaluate the toxicity of AMB emulsion (*In vivo* toxicity studies).

- - 1. **Single dose toxicity in mice:**

It was found that AMB lipid emulsion was less toxic than AMB conventional (AMFOCARE) and Amphotericin lipid complex (ABELCET).

**b) Repeat dose toxicity study in mice**

The AMB lipid emulsion showed less toxicity than AMFOCARE

**c) Single dose toxicity in rats**

The AMB lipid emulsion was less toxic than AMFOCARE.

**d) Repeat dose toxicity study in dogs**

AMB lipid emulsion offers significant protection against hepatotoxicity and nephrotoxicity as compared to Conventional AMB

***In-Vitro* effects of Amphotericin B on Human Cells:**

The *in vitro* toxicity data clearly indicate that AMB emulsion and lipid complex is less toxic than AMFOCARE when the target cells are human RBCs, which is in agreement with reported data (*Tabosa Do Egito, et al, 1996*)

## Clinical Studies

**Amphomul^®^**

A clinical study was carried out in 48 patients at two centers in India comparing safety and efficacy of three different regimens of intravenous AMB emulsion (Amphomul^®^) for treatment naive and treatment resistant cases of Kala-azar.

In this study, three regimens of this formulation were tried viz., 2 mg/kg/day, 3.5 mg/kg/day and 5 mg/kg/day (approximate total dose- 15 mg/kg). The results of this study showed an exceptionally better safety profile and at the same time maintaining a comparable efficacy level in Visceral Leishmaniasis patients.

AMB emulsion did not produce any clinically recognizable hepatic, respiratory or hemodynamic disturbances in any patient. Also significant nephrotoxicity, hypokalemia and hypomagnesaemia that are almost always associated with conventional AMB treatment were conspicuous by their absence.

Overall the new emulsion formulation of AMB demonstrated efficacy similar to lipid/conventional formulations in treatment of Kala-azar. Amphomul^®^ was also found to be safe and well tolerated at doses administered (2, 3.5 and 5, in mg/kg dose) IV on alternate days for three days, with very low incidence of infusion related AEs in patients with Visceral Leishmaniasis.

It also produced excellent cure rates across all three arms, for patients who received the cumulative dose of 15 mg/kg over the specified treatment duration of the previous study.

**Safety Data from previous clinical studies with Amphomul^®^**

More commonly encountered side effects:

Chills, fever, headache, nausea and vomiting, loss of Appetite, diarrhoea, stomach pain, hypokalemia, hypoxia.

Less commonly encountered side effects:

Anaemia, leucopoenia, renal function impairment, respiratory distress, thrombocytopenia, back pain, chest pain, dark urine, jaundice, skin rash, anaphylactic reaction, dyspnoea, hypertension, hypotension, tachycardia, facial edema, hives, itching, skin redness, unusual tiredness, weakness.

The association of the above adverse reactions with AMB emulsion is uncertain.

**AmBisome:-**

Liposomal Amphotericin B is approved in several European countries for primary treatment of Visceral Leishmaniasis, and the U.S. Food and Drug Administration had approved it, recommending a total dose of 21 mg/kg; however, in a pilot trial in Indian Visceral Leishmaniasis, liposomal Amphotericin B (AmBisome) in cumulative doses of 6, 10, and 14 mg/kg cured all 10, 9, and 10 patients, respectively.

In the randomized, double-blind, dose-ranging, multicenter trial, 84 patients with Visceral Leishmaniasis refractory to antimony therapy were administered AmBisome at cumulative doses of 3.75, 7.5, and 15.0 mg/kg for 5 consecutive days. Posttreatment apparent cure and definitive cure were assessed at 2 weeks and 6 months after the end of therapy, respectively. Low-dose liposomal Amphotericin B (AmBisome) given for 5 days can cure most patients with Indian kala-azar. The shortened duration of treatment reduces the usual hospital stay from 5 weeks or more to just 5 days. The shortened hospital stay will further reduce the hospital cost and the person-days lost for both patients and attendants, and it will increase the availability of hospital beds 6- to 7-fold. The decreased number of infusions from 15 or more to 5 reduces the costs of fluids, tubing, and other hospital supplies. Although clinical availability of AmBisome is a major therapeutic advance in the treatment of leishmaniasis, it may remain out of reach of most patients with Visceral Leishmaniasis because of its higher cost (*Sundar S et al, 2002*).

AmBisome at a total dose of 20 mg/kg is safe and effective under routine programme conditions in Bihar. Treatment with a lower dosage would reduce patient hospitalization time by half and save a considerable amount of resources. Various Phase 2 studies using AmBisome at 15 mg/kg have showed good efficacy and safety outcomes, but this dosage has not yet been proven effective under routine programme conditions (*Gupta J et al, 2008*)

## Rationale for the study

Due to the high incidence of drug resistance, drug toxicity and long duration of treatment (required by Sodium Stibogluconate), AMB is increasingly being used as first line treatment of Kala-azar (*Chandra J, 1995*; *Thakur CP, 1998*; *Thakur CP, 2001*). Amphotericin B deoxycholate treatment results in 100% cure, even in Antimony resistant cases (*Thakur CP, 1993*; *Thakur CP, 1999*). Unfortunately, Conventional AMB needs to be administered over 20 to 30 days and causes significant nephrotoxicity (*Thakur CP, 1999*; *Bennet JE, 2001*).

Despite the value of AMB and the importance of early use to prevent emergence of resistant cases and achieve rapid cure, it has often been underutilized in clinical practice. This is because of its unfavorable toxicity profile.

The lipid emulsion form of AMB has been proven to produce dramatically different pharmacokinetic properties. Preclinical and clinical studies have shown it to be better tolerated than conventional drug.

In continuation with Section 9.5 above, it is of obvious scientific interest to further attempt to shorten the duration of treatment with Amphomul^®^. This will reduce the cost of hospitalization and the resulting burden on the healthcare system in developing countries like India. The previous study (BSV-AMBE II-KA-0706) had shown that Amphomul^®^ at the single bolus dose infusion of 15 mg/kg is safe and effective in the treatment of Kala-azar. Amphotericin B emulsion, administered as a 15 mg/kg single bolus dose infusion, had produced excellent initial cure, clinical improvement and definitive cure rates in subjects with treatment naive cases of visceral leishmaniasis (Kala-azar). This study demonstrated higher outcome score of efficacy with Amphotericin B emulsion as a single‑bolus infusion as compared to the study drug given in divided doses. Amphotericin B emulsion did not produce any clinically recognizable hepatic, renal, respiratory or hemodynamic disturbances in any subject. Analysis of vital parameters confirmed absence of any adverse effect of Amphotericin B formulation on hemodynamic parameters at daily doses as high as 15 mg/kg given either on Day 1 as a single-bolus infusion. Overall Amphotericin B emulsion was well tolerated with very low incidence of infusion related AEs.

Hence, on the basis of the results of above Phase II study, Bharat Serums and Vaccines Ltd had decided to go a step forward by doing a prospective, randomized, open-label, multicentric Phase III clinical study of Amphotericin B lipid emulsion. The main aim of the Phase III study will be to demonstrate that low cost, infusion of Amphomul^®^ 15 mg/kg/day is safe and well tolerated in the treatment of Visceral Leishmaniasis (Kala‑azar) as compared to AmBisome.

10. STUDY OBJECTIVES and ENDPOINTS

## OBJECTIVES

### Primary

To compare and evaluate efficacy of Amphotericin B emulsion (15 mg/kg/day as a single dose infusion) compared to AmBisome (15 mg/kg as a single dose infusion) in the treatment of Visceral Leishmaniasis (Kala‑azar)

### Secondary

To assess the safety of infusion of Amphotericin B emulsion (15 mg/kg as a single dose) compared to AmBisome (15 mg/kg as a single dose infusion) in treatment of Visceral Leishmaniasis (Kala-azar).

## ENDPOINTS

### Primary

- Proportion of patients with Amphomul^®^ 15 mg/kg as a single dose infusion achieving
  - Initial cure (splenic aspirate score 0 on Day 30 or on Day 45)
  - Clinical improvement on Day 30 after study drug administration.
    - Absence of fever; and >1 out of following:
      - Increase in hemoglobin concentration by >10% compared to baseline
      - Weight gain compared to baseline
      - Decrease in spleen size by >33% compared to baseline
  - Definitive cure

1. Initial cure at Day 30 or Day 45
2. No signs and symptoms of relapse of Kala-azar for 6 months after study drug administration. If signs and symptoms of relapse of the disease are suspected at any time during the 6 months follow up, splenic aspirate shall be performed at 6 months or earlier after study drug administration.

Proportion of patients achieving all three-efficacy endpoints will be compared across the two groups.

### Secondary

- Incidence of DLTs.
- Incidence of IRTs
- Incidence of nephrotoxicity and hepatotoxicity
- Number of AEs and SAEs
- Laboratory values for different parameters

# 11. STUDY DESCRIPTION (INVESTIGATIONAL PLAN)

## 11.1 Basic Principles and Ethical Considerations

The study will be performed in accordance with the Standard Operating Procedures (SOPs) laid down by SIRO Clinpharm Pvt. Ltd. These SOPs have been developed in accordance with International Conference on Harmonization (ICH) for Good Clinical Practice (GCP); GCPs for Clinical Research in India, 2001; Indian Council of Medical Research (ICMR) Ethical Guidelines for Biomedical Research on Human Participants, 2006 and Schedule Y, Amended 2005 under Drugs and Cosmetics Act and Rules there under, which are consistent with the Ethical Guidelines outlined in Declaration of Helsinki, version 1996 (*Appendix A)*. The study will commence only after the approval of the IRB/IEC.

## 11.2 Sample Size

Considering an estimated difference in proportions of patients achieving definitive cure for Amphomul^®^ vs. AmBisome equals to zero and non-inferiority margin fixed at -0.10, a sample size of 400 patients in ratio of 3:1 in Amphomul^®^ and AmBisome treatment groups (i.e. 300 patients in Amphomul^®^ and 100 patients in AmBisome) is required to assure at least 80% power for the non-inferiority test. Considering a total drop out rate after baseline and major protocol deviations close to 20%, 500 patients (i.e. 375 patients in Amphomul^®^ and 125 patients in AmBisome) will be enrolled in the study.

## 11.3 Study Design

The study will be prospective, two‑arm, randomized, open-label, multicenter, Phase III clinical study. Patients will be randomized to receive infusion of Amphomul emulsion (15 mg/kg as a single dose infusion) administered over 4 to 6 hours or AmBisome 15 mg/kg as single dose infusion over 4 to 6 hours

Only patients for whom the Investigator believes the requirements of the protocol will be complied with should be enrolled in the study.

## Flow of events for the study

##

Signed Informed consent Form,

rK39 dipstick test will be performed prior to any screening procedure Screening

Eligible patients will be randomized to receive EITHER Amphomul® 15 mg/kg as a single dose infusion OR AmBisome 15 mg/kg as a single dose infusion.

Drug will be administered to all the patients on Day 1 over 4 to 6 hours (dose of Amphomul® is 15 mg/kg)

Comparator drug, AmBisome 15 mg/kg administered on Day 1 over 4 to 6 hours.

Patients will be discharged from the hospital on Day 7

Follow-up visit on Day 30: if splenic aspirate score is 0 initial cure achieved, if score is +1 splenic aspiration repeated on Day 45, if the score is above +1 patient considered as treatment failure and discontinued from the study.

At follow-up on Day 45 if splenic aspirate score is 0, initial cure achieved and follow up at 6 months (after the administration of study drug), if the score is +1 and above patient will be considered treatment failure and withdrawn from the study.

End of study visit will be done at 6 months after study drug administration. Definitive cure is achieved if initial cure at Day 30 or 45, followed by no clinical signs and symptoms of Visceral Leishmaniasis at 6 months (after the administration of study drug)

Figure 1: Efficacy and Safety assessment across visits

Period of Hospitalization

Follow up visits

Day 30 Day 45 * 6 months**

Screening Day 1 Day7

V 0 V1 V 2 Additional V3 (EOS)

Visit

*Day 45 Visit is only for patients with splenic aspirate score +1 on Day 30.

**Follow-up visit at 6 months after study drug administration (EOS Visit).

## 11.5 Study Duration, Treatment Interruption and Termination

The clinical part of the study will start after the approval of the responsible IRB/IEC and after the site initiation visit is performed.

The timeframes of the study will be as follows:

- Total duration of the Clinical study: 1 year 8 months
- Total Clinical Duration (maximum) per patient: 7 months
- Recruitment Period (all patients): 7 months
  - - - **Study Treatment Interruption:**
- In case of infusion related reactions temporary discontinuation of the infusion or administration of concomitant medications as judged appropriate by the investigator, (other than prohibited medications) will be permitted.
- None of the patients will be replaced.

**The entire study may be prematurely terminated/discontinued** if there is,

- Comprehensive deficiency in the recorded data or protocol compliance so that the results of the study cannot be reliably assessed.
- The Sponsor, PI(s) and the Project Manager, if appropriate commonly decide premature termination, after consultation with the Ethics Committee and the study sponsor.

In the event of early termination, PI(s) will cease use of the IP immediately. All outstanding Case Report Forms (CRFs) will be completed and returned to SIRO together with completed drug inventory, records and remaining trial material.

A written statement fully documenting the reasons for such a termination will be provided from Bharat Serums and Vaccines Ltd. to the IRB/IEC and Drugs Controller General of India (DCGI).

# 12. STUDY POPULATON

## Sample size

Considering a drop-out rate of 20%, 500 patients will be enrolled to get 400 evaluable patients

Considering an estimated difference in proportions of patients achieving definitive cure for Amphomul^®^ vs. AmBisome equals to zero and non-inferiority margin fixed at -0.10, a sample size of 400 patients in ratio of 3:1 in Amphomul^®^ and AmBisome treatment groups (i.e. 300 patients in Amphomul^®^ and 100 patients in AmBisome) is required to assure at least 80% power for the non-inferiority test. Considering a total drop out rate after baseline and major protocol deviations close to 20%, 500 patients (i.e. 375 patients in Amphomul^®^ and 125 patients in AmBisome) will be enrolled in the study.

## Target Population

Patients of either gender, between 5 and 65 years of age (both inclusive) with Visceral Leishmaniasis (Kala-azar).

## Inclusion Criteria

Following patients will be included in the study -

1. Male or female patients aged between 5 to 65 years (both inclusive).
2. Patient/patient’s LAR/impartial witness is willing and able to give written informed consent to participate in the study.
3. Clinical signs and symptoms of Visceral Leishmaniasis (fever of over 2 weeks duration and splenomegaly)
4. Presence of amastigotes (*Leishmania donovani* bodies) at prescreening detected by rK39 dipstick test with confirmation of Kala-azar by splenic or bone marrow aspirate smear examination.
5. No previous treatment for Visceral Leishmaniasis within 30 days from screening.
6. Non-pregnant, non-lactating females of age ≥5 years, and woman of childbearing potential (any woman who has reached menarche) who are willing to use acceptable methods of contraception like long term acting injections ex. Depo‑Provera.
7. Negative UPT in all women physiologically capable of becoming pregnant (any woman who has reached menarche)
8. Hemoglobin ≥5 g/dL.
9. WBC count ≥ 1000/ cmm,
10. Platelet count ≥ 50000/cmm.
11. ALT, AST and alkaline phosphatase ≤2.5 times the ULN.
12. Prothrombin time ≤4 seconds above the control
13. Normal serum potassium levels
14. Negative tests for HIV, HCV and HBsAg

## Exclusion Criteria

Patients who meet any of the following criteria at screening or prior to treatment assignment have to be excluded from study entry:

1. Patients with past history of treatment with Amphotericin B or any other drug for Visceral Leishmaniasis within 30 days prior to screening.
2. Patients positive for HIV, HCV and HBsAg infection, immunocompromised patients (through history).
3. A history or evidence of any concurrent disease that may be serious or life threatening, significant hematological, cardiac, hepatic, renal, respiratory, neurological or metabolic disease or any condition which, in the opinion of the investigator, may constitute a safety concern or interfere with the evaluation of the study objectives or may prevent the patient from the completing study therapy or subsequent follow-up may have a significant impact on the outcome of the study.
4. Concurrent diabetes, tuberculosis or bacterial pneumonia (on chest X-ray, ESR at screening, past history) or any other infectious or major psychiatric disease.
5. Patients with concurrent Malaria will not be included in the trial. Parascreen antigen test for detection of malarial parasites will be performed at screening visit. Patients diagnosed with malaria could be rescreened for enrollment in the trial after adequate treatment of Malaria; & if the parascreen antigen test for detection of malarial parasites is found negative then the patient could be enrolled in the trial
6. History of major surgery within 2 weeks prior to screening.
7. BUN and serum creatinine >1.5 times ULN, and serum total bilirubin >1.5 times ULN
8. Proteinuria ≥2+
9. Pregnant or nursing women.
10. History of alcoholism or illicit drug use/ abuse, or any condition associated with poor compliance.
11. Known hypersensitivity to Amphotericin B, inactive ingredients of Amphomul^®^ formulation, inactive ingredients of AmBisome formulation.
12. Patients receiving any of the medications prohibited by the study protocol.
13. Simultaneous participation in another trial or received any IP <30 days prior to enrolment.
14. Any condition which in the investigator’s opinion may prevent the patient from completing the study therapy or follow-up

## Patient Information and Informed Consent

Written informed consent must be obtained from each patient before enrolling in the study. The method of explanation to the patient/LAR/impartial witness and obtaining their consent should comply with the ICH‑GCP Guidelines, with GCP for Clinical Research in India, 2001; ICMR’s Ethical Guidelines for Biomedical Research on Human Participants, 2006 and Schedule Y, Amended 2005 under Drugs and Cosmetics Act and Rules there under, and the ethical principles in the amended Declaration of Helsinki, whichever represents the greater protection for the individual.

The patient’s/LAR’s signature/thumb impression or Impartial witness’s signature is to confirm and document patient’s willingness to participate after having received the following information:

- The study involves research
- Study objectives
- Effects (including possible side effects and reasonably foreseeable risks or inconveniences caused to the patient*)* of the study drug and/or procedures in the study
- Possible benefits of the study drug
- Performance of the study, duration, allocation to treatment and approximate number of patients involved in the trial, additional and/or invasive procedures that patient needs to undergo
- Patient's responsibilities
- Alternative treatment possibilities
- Voluntary participation in the study and the possibility to refuse or withdraw at any stage without giving reasons and without any disadvantages for the patient
- Possibility regarding further information
- Information that it is not possible to participate in the study if the patient has participated in another clinical study during the course of the past 30 days
- Information that the patient is not permitted to simultaneously participate in another clinical study
- Recording of data and disclosure to the sponsor/sponsor representatives, the IRB/IEC or the regulatory authority
- The records identifying the patient will be kept confidential and will not be made publicly available, to the extent permitted by law.
- Information that if any new information that relates to patient’s decision regarding continuation of patient's participation in the study, if available during the course of study will be made available to the patient/LAR/ impartial witness
- The part of study that is experimental
- Contact for further information and in case of trial related injury
- Any foreseeable reasons when patients' participation in study could be terminated
  at the outset of the study the patient/LAR/impartial witness receives an information sheet (in English or a language which patient understands) containing information about the study on which the patient/LAR must consent by signature/thumb impression and impartial witness must consent by signature (enclosure). The consent form will be kept with the Investigator performing the study. A copy of patient information sheet and signed consent form must be handed over to the patient/LAR/impartial witness.

## Patient Completion and Withdrawal

### For each patient, according to the protocol, the study is deemed

- **Completed**

The patient will be administered study treatment as per the protocol, and completes all the safety and efficacy assessments on- Day 7, Day 30, Day 45 (only for patients with splenic aspirate score +1 on Day 30), and at 6 months after study drug administration.

- **Discontinued/withdrawn**- Withdrawal from the study can occur under the following circumstances:
  - 1. Death
    2. Adverse event
    3. Lack of efficacy (treatment failure)
    4. Patient consent withdrawal
    5. Investigator’s discretion
    6. Sponsor’s decision
    7. Pregnancy in a patient
    8. Lost to follow-up*

*** Lost to follow-up**

Any patient who was lost to follow-up before the 6 months visit will be withdrawn from the study.

### Procedures for handling patients who discontinue the study prematurely

Investigators should make an attempt to contact those patients who do not return for follow-up on Day 30, Day 45 (only for patients with splenic aspirate score +1 on Day 30) and 6 months after study drug administration. Information gathered should be described in the source and CRF.

### Recording Patient Withdrawals

In the event of a patient withdrawal, the following information must be recorded in the CRF:

- Date of withdrawal
- Reason for withdrawal
- Last treatment date
- Follow-up assessments

If a patient withdraws or is withdrawn from the study, he/she will be requested to return for a follow-up visit after 1 week of the withdrawal date, for recording AEs and physical examination.

The Investigator should continue to follow-up all SAEs or other AEs that are considered to be related to the study drugs, until resolved or assessed to be chronic or stable by the Investigator. This should be documented in the patient’s medical records. This follow‑up may be extended beyond the end of the study period.

In the case of patients lost to follow-up, all attempts to contact the patient must be documented in the patient’s medical records.

### Replacement of Patients Withdrawn

- None of the patients will be replaced.

# 13. COURSE OF STUDY

- Screening of patients with rK39 test and splenic aspiration will be performed to ensure that only those patients who have pathologically confirmed Visceral Leishmaniasis are enrolled in the study.
- Patients will be randomized to receive either Amphomul^®^ 15 mg/kg single dose infusion or AmBisome 15 mg/kg single dose infusion on Day 1.
- Patients will be hospitalized for duration of 7 days starting from the first dose of the study drugs.

#### Screening Visit*-*Visit 0 (Day ‑7 to Day 0)

- Screening can be done during Day ‑7 to Day 0 so that the results are available by Day 1 and the patient can be administered the study drug on Day 1.
- The patient/LAR/ impartial witness will be given information on the study and the opportunity to ask for more details. The patient/LAR/ impartial witness will be provided with the written ICF. The Investigator will ask the patient/LAR/ impartial witness whether they agree to participate. Consent will be documented, if given, and will be followed by rest of the screening procedures.
- Detailed physical examination of patients will be performed.
- Prescreening rK39 dipstick test will be done to confirm the presence of amastigotes (*Leishmania- Donovani* bodies), and if positive, this will be followed by splenic or bone marrow aspirate smear examination.
- **rK39 dipstick test:** rK39 dipstick test will be performed prior to any screening procedure The rK39 dipstick test will be performed for serological diagnosis of Visceral Leishmaniasis. This test will be conducted only at screening visit, to avoid unnecessary splenic aspiration. Kalazar Detect™ for Visceral Leishmaniasis-Rapid Test (Manufactured by InBios International, Inc) will be used. The following procedure will be performed.
- 20 μL of serum will be applied to the sample pad.
- Three drops of chase buffer provided will then be applied to the sample pad.
- Results will be read after 10 minutes.
- A red to light pink test line will indicate that the test is positive for Visceral Leishmaniasis. No test line will indicate that the test is negative. In both cases the control line should appear, if the control line is not present the test will be considered as invalid. The figure below shows a test strip that has tested positive for Visceral Leishmaniasis.


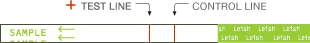


- Only patients who are rK39 positive will be evaluated for inclusion/exclusion criteria, demography and medical history.
- Findings of Visceral Leishmaniasis will be confirmed clinically and documented.
- Baseline laboratory investigations, ECG and chest X-ray will be performed to ensure that all the inclusion criteria are met. Patients will be randomized only after the investigator has assessed them for inclusion and these criteria are all fulfilled
- Detailed systemic examination of patients will be performed.
- Sufficient amount (Approximately 10 mL) of venous blood will be withdrawn, for performing following investigations:
  - Hematology:- hemoglobin, RBC, ESR, platelets, total and differential leukocyte count
  - Biochemistry:-
    - Liver function tests (Serum ALT, AST, alkaline phosphatase, total bilirubin, serum albumin, prothrombin time)
    - Renal function tests (serum creatinine, BUN*)*
    - Electrolytes (sodium, potassium, magnesium)
  - HIV test, HCV and HBsAg tests.
  - Fasting blood sugar test.
  - Parascreen antigen test for detection of malarial parasites
- X-ray chest and 12-lead ECG
- UPT (for females of child bearing potential only)
- Urine analysis (routine, microscopy )
- **Splenic aspirate**
  - Splenic aspirate will be performed to assess parasitological cure.
  - If the spleen is not palpable then Bone marrow aspirate will be done.
  - Splenic aspiration will be done only if rK39 test is positive.
  - Prothrombin time will have to be carried out before splenic aspiration and procedure will be done only if result is ≤4 seconds above the control.
  - Splenic aspirate slides will be stained by either Leishman’s stain or Giemsa stain. Both the stains contain eosin as Acidophilic Dye Component. Leishman’s stain contains Methylene Blue as Basophilic Dye Component whereas Giemsa Stain contains azure B as Basophilic Dye Component. Leishman's Stain is technically easy to perform and procedure of staining takes less time. Parasite density will be graded microscopically (X10 eyepiece X100 objective) using a logarithmic scale from +1 (1 to 10 amastigotes per 1000 oil immersion microscopic fields) to +6 (>100 amastigotes per oil immersion field). The grading will be done as follows:

| **Table 1: Grading system for grading parasite density microscopically** | |
| --- | --- |
| **Grade** | **Average parasite density** |
| +6 | >100 amastigotes/field |
| +5 | 10-100 amastigotes/field |
| +4 | 1-10 amastigotes/field |
| +3 | 1-10 amastigotes/10 fields |
| +2 | 1-10 amastigotes/100 fields |
| +1 | 1-10amastigotes/1000 fields |
| 0 | No amastigotes/1000 fields |

- Previous and concomitant medications will be recorded.
- Data obtained from the laboratory investigations will be used as soon as available to decide patient’s participation in the study.

**Visit 1-(Day 1 to Day 7)**

**Visit 1 - Day 1 (Start of study drug treatment)**

- The patients will be re-evaluated clinically.
- If all eligibility criteria are satisfied, then the patient will be included in the study to receive treatment with Amphomul^®^ or AmBisome.
- Patients not fulfilling any of the eligibility criteria on this visit would not be included in the study and labeled as screening failures.
- Laboratory investigations will be done at Investigator’s discretion.
- Permuted Block randomization with block of size 4 and ratio of 3:1 in the two groups (Amphomul and AmBisome) will be generated for each center. Patients will be randomized in the ratio of 3:1 to receive either Amphomul^®^ infusion (15 mg/kg as a single dose infusion) over 4 to 6 hours or AmBisome 15 mg/kg as a single dose infusion over 4 to 6 hours.
- Test dose consisting of 1 mg will be administered in 5% dextrose over a period of approximately 15-20 minutes prior to administration of full-dose infusion of Amphomul^®^.
- The IP (Amphomul^®^)*,* diluted (maximum dilution allowed to 1 mg/mL) in 5% dextrose will be administered as an infusion.
- Before administration AmBisome has to be diluted with 12 mL sterile water to yield a preparation containing 4 mg of Amphotericin B/mL. This diluted AmBisome will be further diluted with 5% Dextrose to a concentration not less than 1 mg/mL.
- Test dose consisting of 1 mg will be administered in 5% dextrose over a period of 10 minutes prior to administration of full-dose infusion of AmBisome
- No premedication will be administered prior to the study drug administration.
- However, if patient experiences chills or any infusion related reactions after starting infusion, concomitant medication(s) can be administered at the discretion of the investigator.
- As for use of all Amphotericin B products, facilities for cardiopulmonary resuscitation will be kept readily at hand when administering the drug due to possible occurrence of anaphylactic reactions.
- In case of severe hypersensitivity reactions uncontrolled by the premedications or cardiopulmonary complications of hypersensitivity, the patient would be discontinued from the study and would be treated for the complications till the patient becomes stable.
- The patient will be closely monitored for approximately 6 hours following the completion of infusion for any IRTs.
- Vital signs will be recorded.
- After the study drug administraton, patient will be daily monitored for any AEs and SAEs, which will be carefully recorded in the patient’s medical records and CRF.
- Previous and concomitant medications will be recorded.
- DLT assessment will be performed

**Visit 1 - Day 7**

- A thorough clinical assessment through physical examination and vital signs will be performed.
- Safety assessment (AEs, SAEs, IRTs and check for nephrotoxicity and hepatotoxicity and laboratory values for different parameters) will be done.
- Laboratory investigations done:-Hematology, Renal and Liver function tests, electrolytes as outlined in the screening section.
- Monitoring, recording and reporting for AEs, if any, will be done appropriately.
- Previous and concomitant medications will be recorded.
- DLT assessment will be performed
- The patient will be discharged and asked to return on Day 30. A thorough clinical examination will be done.

**Visit 2- Day 30 (+/- 5 days):**

- A thorough clinical examination will be done.
- Splenic aspirate will be done to assess parasitological cure and will be graded in exactly the same manner as outlined above. The score will be assessed as follows:
  - 1. If the score is 0, then it is concluded as Initial cure achieved. Patient will be followed up till 6 months after study drug administration.
    2. If the score is +1 then repeat confirmatory splenic aspirate at 15^th^ day after Day 30 aspirate (i.e. Day 45 of study)
    3. If the score is above +1, then these patients will be considered as treatment failures and will be discontinued from the study & rescue medication will be given. No further follow‑up will be performed.
- Prothrombin time will have to be carried out before splenic aspiration and procedure will be done only if result is ≤4 seconds above the control
- Safety assessment (AEs, SAEs, IRTs, nephrotoxicity hepatotoxicity and laboratory values for different parameters) will be done
- Laboratory investigations done:-Hematology, Renal and Liver function tests, electrolytes as outlined in the screening section
- Vital signs will be recorded.
- Monitoring, recording and reporting for AEs, if any, will be done appropriately.
- Previous and concomitant medications will be recorded.
- Other laboratory investigations can be done, at the investigator’s discretion.

**Visit 2 - Day 45 (Additional Visit, +/-5 days):**

- This visit will be done only for those patients who did not have complete parasitological cure on Day 30 and had a splenic aspirate score of +1. If the score is +1 and above, the patients will be considered as treatment failures and will be discontinued from the study and given rescue medication as deemed fit by the Investigator.
- A thorough clinical examination will be done.
- Splenic aspirate will be done to assess parasitological clearance. The score will be assessed as follows :

1. If the score is 0, then it is concluded as initial cure achieved. Patient will be followed up till 6 months after study drug administration.
2. If the score is +1 or above, then these patients will be considered as treatment failures and will be discontinued from the study and rescue medication will be given. No further follow-up will be performed.

- Prothrombin time will have to be carried out before splenic aspiration and procedure will be done only if result is ≤4 seconds above the control.
- Vital signs will be recorded.
- Patients who have parasitological clearance on Day 30 will not visit on Day 45.
- Laboratory investigations done:-Hematology, Renal and Liver function tests, electrolytes at investigator’s discretion.
- Monitoring, recording and reporting for AEs, if any, will be done appropriately.
- Previous and concomitant medications will be recorded.

**Visit 3 - End of study visit- 6 months after study drug administration (-2 weeks to +4 weeks):**

- A thorough clinical examination will be done.
- Safety assessment (AEs, SAEs, IRTs, check for nephrotoxicity and hepatotoxicity and laboratory values for different parameters) will be done
  - Laboratory investigations will be done:-Hematology, Renal and Liver function tests, electrolytes as outlined in the screening section.
- Splenic aspirate will be done to assess parasitological cure in patients in whom a relapse of the illness is indicated by recurrence of fever, splenic enlargement. In others, only clinical assessment will be done to define definitive cure at 6 months.
- Prothrombin time will have to be carried out before splenic aspiration and procedure will be done only if result is ≤ 4 seconds above the control
- Definitive cure will be defined as initial cure at Day 30 or 45 followed by absence of clinical signs and symptoms of relapse of Kala-azar till 6 months after study drug administration. If clinical signs and symptoms of relapse of the disease is suspected at any time during the 6 month follow up, splenic aspirate shall be done at 6 months or earlier after study drug administration.
- Vital signs will be recorded.
- Monitoring, recording and reporting for AEs, if any, will be done appropriately
- Previous and concomitant medications will be recorded.

**Study Treatment Interruption:**

- In case of any episode of intolerable toxicity or abnormal laboratory findings temporary discontinuation of the study drug as judged by the investigator will be allowed.
- In the event of any emerging complication or deterioration, all possible measures will be taken to safeguard the health and life of a patient, notwithstanding the possibility of dropout from the study.

**DLTs during the study:**

- Recruitment of all subsequent patients in any dosage regimen will be stopped or discontinued if the overall percentage of patients in the study with DLT is >33%. This criterion will only be applicable after randomization of 25^th^ patient in either arm.
- DLT of the study drugs for this study is defined as:

i) Any drug related grade III or higher AE classified as nephrotoxicity/ hepatotoxicity evaluated as per NCI-CTC-AE-v3).

ii) Any other drug related grade IV or higher toxicity except hematological toxicity (evaluated as per NCI-CTC-AE-v3).

iii) If patient’s hematological parameter is in CTC grade III or grade IV at baseline, then a DLT would be considered if there is reduction of ≥33% from baseline.

iv) If patient’s hematological parameter is within normal range or is in CTC grade I or CTC grade II at baseline, then a DLT would be considered if the parameter drops to grade IV or higher

- All the patients will be assessed for DLT up to Day 7 after the administration of first dose of the study drug.

**Treatment strategy during the study:**

The patients will be randomized in the ratio of 3:1 to receive either Amphomul^®^ 15 mg/kg administered as a single dose infusion over 4 to 6 hours or AmBisome 15 mg/kg as a single dose infusion over 4 to 6 hours will be administered on Day 1.

**All the patients recruited will be hospitalized up to Day 7 counting from Day 1 of therapy irrespective of the regimen used.**

# STUDY CALENDAR

Patients **recruited will be hospitalized till Day 7 counting from Day 1 of therapy irrespective of the regimen used.** The evaluation schedules will be as below:

|  | **Screening** |  | | | | **End of Study Visit (EOS)** |
| --- | --- | --- | --- | --- | --- | --- |
| **Visit** | **V0** | **V1** | | **V2** | | **V3** |
| **Days** | **D–7 to**  **D0** | **D1** | **D7** | **D30**  **(+/-5 days)** | **D45***  **(+/- 5 days** | **6 months after study drug administration (‑2 weeks to +4 weeks)** |
| Informed consent | X |  |  |  |  |  |
| Demography and Medical History | X |  |  |  |  |  |
| Systemic Examination | X | X | X | X | X | X |
| Vital signs | X | X | X | X | X | X |
| Eligibility criteria | X | X |  |  |  |  |
| rK39 dipstick test | X |  |  |  |  |  |
| Prothrombin time^##^ | X |  |  | X | X | X |
| Splenic aspirate | X |  |  | X | X | X^###^ |
| HIV test | X |  |  |  |  |  |
| HCV | X |  |  |  |  |  |
| HBsAg | X |  |  |  |  |  |
| Fasting Blood Sugar test | X |  |  |  |  |  |
| Parascreen antigen test | X |  |  |  |  |  |
| Safety  Laboratories | X |  | X | X |  | X |
| X-ray Chest | X |  |  |  |  |  |
| 12-lead ECG | X |  |  |  |  |  |
| UPT(for females of child bearing potential) | X |  |  |  |  |  |
| Treatment Administration |  | X |  |  |  |  |
| DLT Assessment |  | X | X |  |  |  |
| Previous and Concomitant medication | X | X | X | X | X | X |
| Hospitalization |  | X | X |  |  |  |
| Adverse Events | X | X | X | X | X | X |
| *****Visit on Day 45 will be done only for those patients who do not have complete parasitological clearance on Day 30.  ^##^ Prothrombin time will have to be carried out before splenic aspiration and procedure will be done only if prothrombin time ≤4 seconds above the control.  ^###^Splenic aspiration at 6 months will be done only for those patients in whom relapse is suspected. | | | | | | |

# 15. STUDY FORMULATION

## Product information

### 15.1.1 Investigational product (Amphomul^®^)

Amphomul^®^ will be manufactured under Good Manufacturing Practice (GMP) by Bharat Serums and Vaccines Ltd.

Amphomul^®^ will be supplied as a yellowish emulsion for IV injection in 10 mL vials for single use. Each vial contains active ingredients as shown in Table 2.

**Table 2: Composition of Amphomul^®^ per mL of emulsion:**

| Ingredient Amount | |
| --- | --- |
| *Active Ingredient* | |
| Amphotericin B | 5mg |
| *Other Ingredients:* | |
| Soybean oil | 200 mg |
| Glycerin | 22.5 mg |
| Purified Egg lecithin | 12 mg |
| Sodium Hydroxide | q.s. |
| Water for Injection | q.s. |

### 15.1.2 Reference product (AmBisome)

AmBisome will be supplied as 15 mL glass vial (Each vial contains 50 mg Amphotericin B). One pre-packaged, disposable sterile, 5-micron filter will be supplied with this.

**Table 3: Composition of AmBisome per vial:**

| **Ingredient** | **Amount** |
| --- | --- |
| Amphotericin B | 50 mg |
| Hydrogenated soy phosphatidylcholine | 213 mg |
| Cholesterol | 52 mg |
| Distearoylphosphatidylglycerol | 84 mg |
| Alpha tocopherol | 0.64 mg |
| Sucrose | 900 mg |
| Disodium succinate hexahydrate | 27 mg |

## Supply, storage and accountability of study drug

Study drug will be provided by Bharat Serums and Vaccines Ltd. /SIRO for the requested number of patients from time to time depending upon the number of patients likely to be recruited.

### Drug storage

All vials must be stored in a safe and locked place with no access for unauthorized personnel. They must be stored below +25°C and must not be frozen. Storage temperature should be monitored once a day approximately at similar time everyday in a study-specific temperature log provided by SIRO.

In case of AmBisome: the reconstituted product concentrate may be stored for up to 24 hours at +2° to +8° C (36° -46° F) following reconstitution with sterile water for injection, USP. Injection of AmBisome should commence within 6 hours of dilution with 5% Dextrose Injection.

In the event of disruption of the cold chain or accidental freezing, the drugs should not be administered and the Investigator or designee should contact SIRO immediately to receive further instructions and/or fresh supplies.

### Drug supplies

The investigator will be supplied with enough doses of study drugs to administer them as daily IV infusions to each patient as described in the present protocol. An additional 5% of their respective amounts will be supplied for replacement in case of breakage or inappropriate storage conditions.

Amphomul^®^ will be supplied in 10 mL vials, each containing Amphotericin B 5 mg/mL emulsion.

AmBisome will be supplied in 15 mL glass vial each containing 50 mg of Amphotericin B.

In case the container of study drug is broken or unusable, the Investigator should replace it with a fresh vial. Although the Sponsor need not be notified immediately in these cases, documentation of the use of another vial and reason for using it must be recorded by the Investigator on the drug accountability form.

### Drug accountability

The Investigator must acknowledge receipt of study drug supplies for each shipment received.

A drug accountability log should be maintained by the staff responsible for storage/administration of the study drugs to the patient. This should record which supplies are issued to which patients. All times the figures of supplied, used and remaining drug vials should match. All used vials must be kept until the study monitor performs drug accountability and retrieves them.

The drugs supplied for this study are only intended for use in the patients in this study. They must not be diverted for use elsewhere.

The study monitor should be notified immediately of details of any supplies which are inadvertently damaged. Details of any supplies (which are inadvertently damaged or unaccountable for any reason) should be given on this log, which will be collected by the study monitor at the end of the study. At the end of the study, it must be possible to reconcile delivery records with those of used and unused stocks. Account must be given of any discrepancies.

Both used and unused drug vials should be sent back to SIRO for destruction in accordance with provided instructions.

## Randomization and Blinding

- Since this is an open label study, blinding is not applicable.
- Permuted Block randomization schedule with block of size 4 and ratio of 3:1 in the two groups (Amphomul and AmBisome) will be generated for each center.
- Treatment with IP/Comparator drug will begin only after successful screening (signing informed consent and fulfillment of all eligibility criteria).
- After meeting eligibility criteria, the patient will sequentially be allotted to unique subject ID and treatment as per randomization schedule for that center.
- A separate screening and enrolment log will be maintained.
- Neither the Investigators nor patient/patient’s LAR will be blinded to the treatment regimen.

## Code Handling and Breaking

In this study since there will be no blinding, code handling and breaking is not applicable.

## Packaging and Labeling of Study drug

Amphomul^®^ will be packed in a carton labeled with at least the following information according to GMP guideline Volume 4, Annex 13 and the local law: protocol number, drug name, route of administration, storage, lot no., expiry date, “STRICTLY FOR CLINICAL TRIAL USE ONLY”.

The outer big white carton, in turn, will contain small manufacturer-provided boxes containing glass vials of Amphomul^®^*.*

##### Original label for Amphomul^®^ vial

Each vial of Amphomul^®^, in addition to its original print provided by the manufacturer/ Sponsor bearing the manufacturer’s name, formulation details, batch number and manufacturing/expiry dates, will bear a label with free space for the Investigator sites to enter the following details for each patient:

a) Patient Number

b) Vial number

c) Date of use

This will be provided in **triplicate**– one tear-off label to be stuck on the medication log, one to be stuck on the CRF and the other to remain on the vial.

**Packaging and labeling of AmBisome**

AmBisome for Injection is available as single 50 mg vial cartons and in packs of ten individual vial cartons. Each carton contains one pre-packaged, disposable sterile 5 micron filter.

Each vial of AmBisome, in addition to its original print provided by the manufacturer/ Sponsor bearing the manufacturer’s name, formulation details, batch number and manufacturing/expiry dates, will bear a label with free space for the Investigator sites to enter the following details for each patient:

a) Patient Number

b) Vial number

c) Date of use

This will be provided in **triplicate**– one tear-off label to be stuck on the medication log, one to be stuck on the CRF and the other to remain on the vial.

## Dosing schedule and Administration

### Investigational product (Amphomul^®^)

- Amphomul^®^ shall be administered by IV route at the site of appropriate IV access. It can also be administered into the central venous catheters, if appropriate. The drug should be administered as a slow IV infusion.
- The Investigator or designee will be required to withdraw Amphomul^®^ from the vial(s), and administer each patient the dose specified as slow IV infusion.
- Before administration, Amphomul^®^ will be diluted to 1 mg/mL in 5% dextrose.
- Test dose consisting of 1 mg will be administered in 5% dextrose over a period of approximately 15-20 minutes prior to administration of full-dose infusion of Amphomul^®^.
- The IP (Amphomul^®^)*,* diluted (maximum dilution allowed to 1 mg/mL) in 5% dextrose will be administered as a slow IV infusion over 4 to 6 hours.
- As for use of all Amphotericin B products, facilities for cardiopulmonary resuscitation will be kept readily at hand when administering the drug due to possible occurrence of anaphylactic reactions.
- The patients will be observed for 6 hours following the end of infusion, with appropriate medical treatment readily available in case of a rare anaphylactic reaction during the administration of infusion. Reactions that occur during this time must be recorded by the Investigator in the CRF.
- In case of any toxicity due to study drug, patient will be withdrawn from the study as per investigator’s discretion

### Reference product (AmBisome)

- AmBisome shall be administered by IV route at the site of appropriate IV access. An in-line membrane filter may be used for the IV infusion of AmBisome; provided the mean pore diameter of the filter is not less than 1.0 micron. The drug should be administered as a slow IV infusion.
- The Investigator or designee will be required to withdraw AmBisome from the vial(s), and administer each patient the dose specified as slow IV infusion.
- Before administration AmBisome has to be reconstituted with 12 mL sterile water to yield a preparation containing 4 mg of Amphotericin B/mL. This reconstituted AmBisome will further be diluted with 5% dextrose to a concentration not less than 1 mg/mL.
- Test dose consisting of 1 mg will be administered in 5% dextrose over a period of 10 minutes prior to administration of full-dose infusion of AmBisome.
- AmBisome diluted in 5% dextrose (to a concentration not less than 1 mg/mL) will be administered as a slow IV infusion over 4 to 6 hours.
- As for use of all Amphotericin B products, facilities for cardiopulmonary resuscitation will be kept readily at hand when administering the drug due to possible occurrence of anaphylactic reactions.
- The patients will be observed for 6 hours following the end of infusion, with appropriate medical treatment readily available in case of a rare anaphylactic reaction during the administration of infusion. Reactions that occur during this time must be recorded by the Investigator in the CRF.

### Premedication

Patients will not be given any premedications prior to infusion of study drugs. However, in case of infusion related events during the infusion of the study drug and if considered necessary by the investigator appropriate concomitant medications will be administered.

### Duration and Interruption of study drug treatment

In case of any intolerable toxicity or abnormal laboratory findings temporary discontinuation of the study drug as judged necessary by the investigator, will be permitted.

## Medication Compliance

Since study drug will be administered by the investigator-team personnel, all patients who complete the study as per protocol will qualify for 100% compliance.

Patients, who do not complete the drug administration schedule as mentioned in the protocol, will be considered non-compliant for the purposes of this study.

# 16. CONCURRENT THERAPY

At each study visit, the PI should question the patient/LAR/impartial witness about any medication taken. Concomitant medications are not expected to interfere with study drug administration during the study, unless they lead to clinically significant impairment of the renal function (nephrotoxic drugs such as aminoglycosides).

In case of any IRTs the patient would be administered Tab Acetaminophen 500 mg or Tab Pheniramine 25 mg or injection paracetamol/pheniramine maleate (or any other concomitant medication as per investigator’s discretion) as concomitant medications.

In case of severe hypersensitivity reactions uncontrolled by the above mentioned medications or cardiopulmonary complications of hypersensitivity, the patient would be discontinued from the study and would be treated for the complications appropriately.

Any other concomitant medication administered must also be recorded in the CRF with generic name and/or trade name of the medication, start and end dates of treatment.

## Concurrent Medication

### Prohibited Medication

1. Any other investigational drugs during the entire study duration
2. Antileishmanial drugs other than study drug during entire study duration.
3. Corticosteroids
4. Skeletal muscle relaxants
5. Cyclosporin
6. Digoxin
7. Vancomycin
8. Aminoglycosides
9. Antifungal
10. Immunosuppresive agents
11. All potentially nephrotoxic drugs should be used with caution.

### Permitted Medications

Medications for other concomitant conditions/diseases

Antibiotics, drugs for restoring cardiorespiratory, metabolic and other systemic functions, premedications, total parenteral nutrition and any blood or platelet transfusion used will be properly documented.

## Rescue Medication

- In the event of any emerging complication or deterioration, all possible measures should be taken to safeguard the health and life of a patient, notwithstanding the possibility of dropout from the study.
- The rescue medication would be AmBisome in case the patient develops worsening of symptoms of Kala-azar, and investigator decides to use an alternative antileishmanial drug, and the patient will be discontinued from study.
- A Phase 2 study was conducted to assess the safety and effectiveness of AmBisome as the first line treatment in 250 patients with primary Visceral Leishmaniasis. Except for a few AEs including lip swelling, urticaria, abdominal and back pain etc AmBisome was found to be safe and effective in a total dose of 20 mg/kg in the treatment of these patients. (*Gupta J et al, 2008*). Hence the dose of AmBisome to be used as a rescue medication in this study will be as follows:
- The rescue medication AmBisome may be given in the dose of 5 mg/kg IV on Days 1, 3, 5 and 7.

# 17. ASSESSMENT OF EFFICACY

**Efficacy:** Patients will be assessed for initial cure on the basis of splenic aspirate score at Day 30 after first dose of the study drug as follows:

| **Splenic aspirate score** | **Interpretation and action to be taken** |
| --- | --- |
| 0 | At Day 30:  Initial cure achieved; Follow-up till 6 months after study drug administration |
| +1 | At Day 30:  Repeat confirmatory splenic aspirate will be done at 15^th^ day after Day 30 aspirate (i.e. Day 45 of study)  At Day 45:  0 ‑ Initial cure achieved, follow-up till 6 months after study drug administration  1+ and above - The patient will be considered as treatment failure and will be withdrawn from study and given rescue medication as deemed fit by the PI. No further follow-up will be performed. |
| above +1 | At Day 30:  These patients will be considered as treatment failures and will be discontinued from the study and given rescue medication as deemed fit by the PI. |

## Efficacy endpoints

- Proportion of patients with Amphomul^®^ 15 mg/kg as a single dose infusion on Day 1 achieving
  - Initial cure (splenic aspirate score 0 on Day 30 or on Day 45)
  - Clinical improvement on Day 30 after study drug administration
  - Absence of fever; and >1 out of following:
    - Increase in hemoglobin concentration by >10% compared to baseline
    - Any weight gain compared to the baseline visit
    - Decrease in spleen size by >33% from baseline
  - Definitive cure:

1. Initial cure at Day 30 or Day 45
2. No clinical signs and symptoms of relapse of Kala-azar for 6 months after study drug administration. If clinical signs and symptoms of relapse of the disease is suspected at any time during the 6 month follow up, splenic aspirate shall be performed at 6 months or earlier after study drug administration.

Proportion of patients achieving all three-efficacy endpoints will be compared across the two groups.

## Efficacy evaluation

- Patient will be considered eligible for efficacy evaluation if study drug has been administered and at least one efficacy assessment has been done.
- Failure of treatment is defined as the presence of any of the following conditions:

Presence of amastigotes on splenic smear examination

Treatment discontinuation due to intolerance

- Patients who discontinued the study drug before completing the total dosage due to a DLT will be considered non-evaluable.
- Non-evaluable patients who receive any dose of study drug will be included in the safety analysis.

Clinical status will be graded on a daily basis until the patient is hospitalized and at each visit thereafter.

- - - Timing of assessments: (applying the criteria for efficacy endpoint)

First assessment will be done at Day 30 after study drug administration

Second assessment on Day 45 ‑(only for patients who have a splenic aspirate score of +1 on Day 30) if the splenic score is 0, follow-up will be performed at 6 months after study drug administration and if the splenic score is +1 and above, no further follow-up will be performed.

If the splenic score is above +1 on Day 30 or Day 45 patient will be a treatment failure and no further follow-up will be performed.

At the end of 6 months after study drug administration, only if relapse is suspected – EOS visit.

# 18. ASSESSMENT OF SAFETY

The recording of AEs is an important aspect of study documentation. It is of utmost importance that all staff members at the site involved in the study are familiar with the content of this section in order to document all AEs according to the detailed guidelines set out below.

## Definitions

### Adverse Events

An AE is any untoward medical occurrence in a patient administered a pharmaceutical product and which does not necessarily have a causal relationship with the treatment. An AE may be any unfavorable symptom, sign, syndrome or disease or even a clinically significant abnormal laboratory value, which occurs for the first time during the course of a study having been absent at baseline or, if present, worsens from baseline, regardless of the suspected cause of the event.

Additionally, they may include signs/symptoms resulting from drug overdose, drug withdrawal, drug abuse, drug misuse, drug interactions, and drug dependence.

### Adverse Drug Reactions

All noxious and unintended responses to a medicinal product related to any dose should be considered Adverse Drug Reactions (ADR). The phrase “responses to a medicinal product” means that a causal relationship between a medicinal product and the adverse event is at least a reasonable possibility, i.e., the relationship cannot be ruled out.

### Serious Adverse Events

An AE will be labeled to be SAE if it-

- Results in death
- Is life threatening
- Requires in patient hospitalization
- Prolongs existing hospitalization
- Results in persistent or significant disability/incapacity
- Is a congenital anomaly/birth defect
- Amounts to any ‘other significant clinical/laboratory event’ of major concern in the opinion of the Investigator

Examples of ‘other significant medical events’ that should be considered serious for the purposes of this study include, but are not limited to, severe early onset reaction such as anaphylaxis, vasovagal episodes, episodes of hypotonia, hyporeactivity or hyperventilation, convulsions etc.

### Unexpected/ Unlisted Adverse Event

Any AE, which is not described in the IB or whose nature, intensity or frequency, differs from what has been described in the IB or a novel event of novel nature will be considered to be an unexpected event.

### Expedited Safety Report

Any AE, which is serious, unexpected and at least possibly related to the IP qualifies for expedited reporting to EC and regulatory authorities.

## Adverse Event Collection Period and Follow up

### Start of AE collection period

Reporting of AEs for any patient will begin after administration of the study drug.

### End of AE collection period

Any AE occurring after the patient has received study drug and until the patient has stopped study participation at the last visit (6 months after study drug administration) will be reported and recorded on CRF

### Follow Up of Adverse Events

After the initial AE/SAE reports, the Investigator is required to follow-up proactively with each patient and provide further information to SIRO safety team regarding the patient’s condition.

During the study, all AEs should be followed up to the event resolution or until the patient’s condition stabilizes or the patient is lost to follow-up.

If any AE is present when a patient completes the study or if a patient is withdrawn from the study, the patient will be re-evaluated within 2 weeks. If the AE has still not resolved at this time, additional follow-up will be performed, as appropriate.

The Investigator should continue to follow-up every SAE or other AE that is considered to be related to the study drugs, until it is resolved or assessed to be chronic or stable by the Investigator. This should be documented in the patient’s medical records. This follow-up may be extended beyond the end of the study period.

## Recording of Adverse Events

### General

AEs will be assessed by the Investigator throughout the study period, during each daily visit by doing clinical examination, review of the laboratory investigations and by directly questioning the patient/patient’s LAR/impartial witness.

All AEs must be noted in the patient’s medical records and AE section of the patient's CRF daily during the study drug administration period, and till the EOS visit (6 months after study drug administration) after study completion/discontinuation/withdrawal.

If known, the diagnosis should be recorded in preference to the listing of individual signs and symptoms. As far as possible each AE should be described to include-

- duration (start and end dates)
- severity/intensity (as pert NCI-CTC criteria)
- relationship to IP (unrelated, unlikely, possibly, probably, definitely)
- influence on further administration of IP
- specific therapy
- results and consequences
- seriousness

AEs already documented in the CRF, e.g. at a previous assessment and designated as ‘ongoing’ should be reviewed at subsequent visits, as necessary. If these have resolved, the documentation in the CRF should be completed.

### Assessment of intensity

The intensity of all unexpected AEs occurring during the course of the study will be graded as follows:

##### Table 4: Assessment of intensity

| **Grading** | **Description of Intensity Grading** |
| --- | --- |
| Mild (Grade 1) | - Barely noticeable and easily tolerable - Does not interfere with functioning, Relieves without medication |
| Moderate (Grade 2) | - Sufficiently discomforting, interrupts routine activity - Symptoms have to be treated but Patient continues in the study |
| Severe (Grade 3) | - Causes severe discomfort interferes considerably with daily activities - Symptoms definitely need medical treatment, patient may not be able to continue in the study |
| Life-threatening (Grade 4) | - May lead to death, if not treated vigorously |
| Death (Grade 5) | - Death due to the AE *per se* |

### Assessment of Causality

Every effort should be made by the PI to explain each AE and assess its causal relationship, if any, to administration of the study drug.

All expected reactions would be considered related to study drug administration.

Causality of all other AEs should be assessed by the PI depending upon various factors like the temporal relationship of the AE to study drug administration, whether AE follows a known pattern of response to IP, any other cause to which the AE can be attributed or intensity of AE changes on rechallenge or dechallenge.

**Table 5: Assessment of causality of AEs**

| **NOT RELATED** | Unrelated | An AE with an incompatible temporal sequence to study drug administration and which could be explained by the patient’s clinical state or other drugs/disease states; in other words, there is no association with the study drug | |
| --- | --- | --- | --- |
|  | Unlikely | An AE with a temporal sequence to study drug administration, which makes a causal connection improbable, and which could plausibly be explained by underlying disease or other drugs/disease states |  |
| **RELATED** | Possible | An AE with a reasonable temporal sequence to study drug administration, but which could also be explained by the patient’s clinical state or other drugs/disease states | |
|  | Probable | An AE follows a reasonable temporal sequence from the time of study drug administration and is unlikely to be attributed to the patient’s clinical state or other drugs/disease states |  |
|  | Definite | The AE follows a reasonable temporal sequence from the time of study drug administration and cannot be explained by the patient’s clinical state or other drugs/disease states |  |

However, failure to assess an exact causality for an AE which is serious and initially assessed as “unclassifiable” within a period of 14 days following initial notification of an SAE to SIRO will result in the event to be deemed as causally related to the IP by default.

### Assessment of outcome

An AE can reach one of the following outcomes:

- Complete recovery from the AE
- Recovered with sequelae
- Ongoing
- Death
- Unknown

### Handling of Unresolved AEs

- If any AE is present when a patient completes the study or if a patient is withdrawn from the study, the patient will be re-evaluated within 2 weeks. If the AE has still not resolved at this time, additional follow-up will be performed, as appropriate.
- Amongst the AEs ongoing on the last study visit as per the study protocol, all the AEs that are serious or the ones, which are related to the IP and are unstable in nature, have to be recorded in the AE form
- The investigator assesses the status of the AEs.
- The AEs recorded at the time of exit are to be followed until,
  - The AEs are considered to become stable as per the investigator’s discretion, or
  - The AEs are no longer considered to be medically significant by the investigator, or
  - The database of the study is declared to be locked.
- The sponsor reserves the right to ask further information on any AE/SAE, which may be considered of interest.

## Reporting of SAEs

- Any SAE occurring during the course of the study should be treated by established standards of care to protect the life and health of the patient.
- If such treatment constitutes a significant deviation from the protocol, the Investigator should notify SIRO immediately to determine whether the patient should be dropped out from the study.
- Any SAE, irrespective of causality, must be **immediately** notified to the safety team at SIRO Clinpharm Pvt. Ltd. **by telephone (+91-22-2584 8429, 24 hour*‑*contact - 9833183195).**
- This must be followed up **within 24 hours by facsimile *(+*91-22-2584 8283*)*** on the study specific SAE reporting form with as much detailed information regarding the event that is available.
- Investigators should not wait to receive additional information to fully document the event, before notifying SIRO of a SAE.
- The SAE reporting form should detail all relevant aspects of the AEs in question. Where applicable, information from relevant hospital case records and autopsy reports should be obtained and provided, in a blinded fashion, to SIRO.
- All SAEs should be reported within 24 hours to SIRO and, within 7 calendar days to IRB/IEC on Investigator’s knowledge of the event. Confirmation of submission to the IRB/IEC must be forwarded to SIRO.
- The event must also be recorded on the CRF AE page.

## Expedited Safety Reporting and Periodic Safety Update Reports

Any serious AE, which is unexpected and at least possibly related to the IP calls for expedited reporting to regulatory authorities (DCGI) and the IECs*/*IRBs of the sites participating in Amphomul^®^ study subject to following timelines:

- 7 calendar days for SAEs involving death and life threatening events
- 14 calendar days for SAEs involving hospitalization or prolongation of hospitalization or persistent or significant disability/incapacity or congenital anomaly/birth defect or any other significant clinical/laboratory event of major concern in the opinion of the Investigator.

As SIRO acts on behalf of the Sponsor, the regulatory reporting time clock starts when any member of SIRO safety team becomes aware of an SAE.

All other SAEs do not warrant expedited reporting to the regulatory authorities and will be informed to the above regulatory authorities and IECs/IRBs for the study sites as a part of Periodic SAE listing at 6 monthly intervals, beginning from the time the first patient is enrolled in the study at any participating site.

All Expedited Safety Reports and Periodic SAE listings of the sites participating in the Amphomul^®^ study should be submitted within 24 hours of Investigator’s receipt of the documents and confirmation of submission to the IRB/IEC must be forwarded to SIRO.

## Protocol Specific safety issues

- All AEs during the study will be graded as per NCI-CTC-AE‑v3.
- Laboratory investigations pertaining to safety analysis (hematological, biochemical and serological and urinalysis*)* will be done from screening visit onwards after starting treatment with Amphomul^®^ as mentioned in Section 14. Study Calendar.
- The evaluating parameters of safety criteria are:
  - Physical findings such as body temperature, weight, rigors/chills, nausea, vomiting, dyspnoea, hypotension, hypertension, tachycardia and any other significant clinical finding.
  - Laboratory parameters as mentioned above.
- The safety criteria shall be evaluated with the definition of change in the laboratory parameters.
- All AEs will be carefully evaluated, recorded, and reported throughout the study.

**Safety criteria (endpoints)**

- Incidence of DLTs
- Incidence of IRTs
- Incidence of nephrotoxicity and hepatotoxicity
- Incidence and frequency of AEs and SAEs (including laboratory abnormalities)

**Nephrotoxicity**

- This would be the main criterion for the analysis of safety and would be defined as grade 3 or higher increase in serum creatinine level, which will be considered as a DLT.
- However, non-DLT rise of >2 times baseline, or, a value >2 mg/dL, whichever is more, shall also be considered for overall evaluation of nephrotoxicity due to the study drug.
- Serum creatinine shall be monitored for this purpose on Day 7 of hospitalization and during subsequent visits till the end of first month.

**Infusion related toxicities (IRTs)**

- Infusion related events would be monitored for approximately 6 hours following the initiation of infusion.
- Following criteria will be carefully evaluated and recorded on all days starting from Day 1 till last day of IP administration as per NCI-CTC AE version 3:
- Fever (rise in oral temperature>1°C above baseline)
- Chills and rigor
- Nausea, vomiting
- Dyspnoea
- Hypotension/hypertension
- Tachycardia

**Other AEs**

Other AEs would be appropriately graded based on the NCI-CTC-AE-v3 criteria. These would be statistically analyzed to know the safety of Amphotericin B emulsion compared to AmBisome along with the following categories -

- Incidence of AEs by preferred term (used from MedDRA)
- Incidence of AEs by system organ class
- Incidence of related/unrelated AEs
- AEs irrespective of causality
- Number of patients experiencing an AE at least once
- Display of severe AEs

**Events NOT to be reported as AEs**

- Progression of underlying disease/study indication/lack of efficacy of the IP should NOT be reported as an AE/SAE.

## Exposure *in utero*

- **Pregnancy during a clinical trial is required to be reported for:**
  - - Female patients exposed to an IP or
    - Partners of male patients participating in the study
  - In event of a pregnancy during the study, the same is required to be reported to the SIRO safety team **immediately via telephone** and in the form of **Pregnancy Report Form (PRF) within 24 hours from the knowledge** of the investigator about the pregnancy.

**A Pregnancy Outcome form** **(POF)** will then be sent to the site from SIRO, which should be filed and a copy of which will be faxed back to the SIRO safety team **within 24 hours** from the knowledge of the pregnancy reaching its outcome.

- - Pregnancy during a clinical trial itself is **not regarded as an AE/ SAE unless**
    - There is a suspicion that an IP may have interfered with the effectiveness of a contraceptive medication.
    - The concerned pregnancy results in one of the following outcomes:

1. Miscarriage/late spontaneous abortion
2. Stillbirth
3. Congenital Malformation/Anomaly
4. Neonatal death *(*within 28 days from birth)
5. Infant death *(*after 28 days from birth but assessed as related to the IP by the Investigator)
   - In case for any pregnancy reported during a clinical trial, if the IP is suspected to have interfered with the effectiveness of a contraceptive medication and/or results in one of the above outcomes, the investigator is required to fill the AE form and the corresponding SAE form patient to the same timelines as mentioned above.

# 19. BLOOD/TISSUE SAMPLE HANDLING AND ANALYSIS

**Laboratory assays**

- Approximately 10 mL of blood will be taken by venous puncture on the days specified in the study calendar. It will be appropriately divided and transferred to respective containers meant for biochemical, microbiological and serological investigations, as stated in the protocol.
- Splenic aspirate
  - Splenic aspirate smear examination (with grading of parasite density) will be performed before initiating treatment with the study drug. However, the test will not be repeated if, Kala-azar has been diagnosed before starting the IP, either by splenic aspirate or bone marrow aspirate smear by the same laboratory (which will be used in this study) and has not been followed by any therapy.
  - Prothrombin time will have to be carried out before splenic aspiration and procedure will be done only if the result is ≤ 4 seconds above the control.
  - Splenic aspirate slides will be stained by either Leishman’s stain or Giemsa stain. Both the stains contain eosin as Acidophilic Dye Component. Leishman’s stain contains Methylene Blue as Basophilic Dye Component whereas Giemsa Stain contains azure B as Basophilic Dye Component.  Leishman's Stain is technically easy to perform and procedure of staining takes less time. Parasite density will be graded microscopically (X10 eyepiece X100 objective) using a logarithmic scale from +1 (1 to 10 amastigotes per 1000 oil immersion microscopic fields) to +6 (>100 amastigotes per oil immersion field).
    - Timings of laboratory investigations would be as mentioned in Section 14 Study Calendar.
    - Any additional/unscheduled investigation can be done at the investigator’s discretion as needed.
    - These investigations, as deemed necessary will also be repeated on the EOS Visit.
    - All blood and body fluids/ tissue samples will be analyzed by the concerned local laboratory.
    - Quality control of slides of 10% of patients will be done at independent laboratory. (at screening as well as initial cure)
    - Window periods of ±5 days will be acceptable for the investigations being done at Day 30 and Day 45. Window period of -2 weeks to +4 weeks will be accepted for investigations being done at the EOS visit.
    - If blood samples from the patients are not drawn at the protocol-defined intervals, and in accordance with the methodology set in this protocol, the patient will not be considered as protocol compliant. However, given the study indication and underlying disease, this will not be reported as deviation of the protocol.

# 20. STATISTICAL ANALYSIS PLAN

## Analysis Sets

For this study, SIRO will consider the following analysis sets:

- **Intent‑to‑treat (ITT) Population:** All patients who received at least one dose of study drug.
- **Modified‑intent‑to‑treat (MITT) Population:** All patients who received study drug as per the protocol‑specified duration and had at least one efficacy assessment throughout the study.
- **Per Protocol (PP) Population:** All patients who complete the study visits per protocol without major protocol deviations.

## Statistical Analysis

### Demographic Data

Measures of central tendency for various demographic variables (age, height, weight etc.) will be provided. Other baseline data including laboratory evaluations and medical history will be tabulated.

### Efficacy Analysis

**Efficacy Endpoints**: Proportion of patients achieving

- Initial cure (splenic aspirate score on Day 30 and 45)
- Clinical improvement on Day 30 after first dose of study drug
  - Absence of fever; and >1 out of following:
    - Increase in hemoglobin concentration by ≥10% compared to baseline
    - Weight gain compared to baseline visit
    - Decrease in spleen size by >33% compared to baseline
- Definitive cure:

1. Initial cure at Day 30 or Day 45
2. No clinical signs and symptoms of relapse of Kala-azar for 6 months after study drug administration. If clinical signs and symptoms of relapse of the disease is suspected at any time during the 6 month follow up, splenic aspirate shall be performed at 6 months or earlier after study drug administration.

Proportion of patients achieving all three-efficacy endpoints will be compared across the two groups.

**Primary Efficacy Analysis:**

Non-inferiority will be assessed by looking at the lower end of a two sided 95% confidence interval (CI) of the difference Ptest - Pref (the difference in the proportions of patients achieving definitive cure in Amphomul^®^ (Ptest) and AmBisome (Pref). Non-inferiority will be only accepted if the lower limit of the two-sided 95% CI is greater than the non-inferiority margin of -0.10.

Non-inferiority will be tested for both MITT and PP population.

**Secondary Efficacy Analysis**:

The proportions of patients achieving initial cure, clinical improvement in both treatment arms will be tabulated along with two-sided 95% confidence interval for both MITT and PP population

### Safety Analysis

Safety Endpoints:

- - Incidence of DLTs
  - Incidence of IRTs
  - Incidence of nephrotoxicity and hepatotoxicity
  - Incidence and frequency of AEs and SAEs (including laboratory abnormalities)

The safety analysis will be performed on the ITT population. Proportion of patients experiencing DLTs, IRTs, nephrotoxicity, hepatotoxicity, AEs and SAEs will be tabulated. Laboratory data will be summarized and displayed, including abnormal values judged to be clinically significant.

SAE reports will be presented individually. AE data will be listed individually, coded using Medical Dictionary for Regulatory Activities (MedDRA) enhanced version. Each AE will be counted only once for a given patient. If the same AE occurs on multiple occasions, the highest severity and relationship will be assumed. If two or more AEs are reported as a unit, the individual terms will be reported as separate experiences.

Concomitant medications will be coded by using the World Health Organization Drug Dictionary (WHODD) Reference List enhanced version.

Vital sign data, physical examination results, ECG examinations results will be summarized for ITT population

### Statistical Analysis of Data

SIRO Clinpharm Pvt. Ltd will perform the statistical evaluation using appropriate statistical tests. Statistical analysis will be performed using SAS^®^ software (SAS Institute Inc., NC, USA).

# 21. PROTOCOL MODIFICATIONS, DEVIATIONS and AMENDMENTS

The Sponsor may at any time modify the Protocol, after approval by Investigator and, if required, by IRB.

## Deviations for a particular patient

When a deviation from the protocol is deemed necessary for an individual patient, the Investigator must contact one of the monitors. Such contact with the monitor must be made as soon as possible to permit a decision as to whether or not the patient is to continue the study; any departure from the protocol will be authorized only for that patient. In the event of any drug-related toxicity, all possible measures should be taken to protect well being and life of the patient, notwithstanding the possibility of dropout from the study and regardless of contact with/permission from any other member involved in the trial. A description of the departure from the protocol and the reason(s) for it must be recorded in the CRF for such patients.

## Amendments, if any for the study

### Administrative modifications

Administrative or technical modifications (like change in monitor or telephone numbers), which do not interfere with the patient's health interests will be in writing and filed as amendments to the protocol. The IEC/IRB has to be informed by the PI regarding every such amendment.

### Clinical modifications

Modifications, interfering with the patient's health interests and involving changes in the design of the study or its scientific significance require a new approval of the IEC/IRB. The Sponsor and investigator will agree to implement/adhere to such modifications.

# 22. ADMINISTRATIVE MATTERS- ADHERENCE TO GOOD CLINICAL PRACTICE (GCP) GUIDELINES

## Regulatory requirements

DCGI acts as the nodal office for approval of clinical trial applications. The study will be started only after notifying DCGI.

## Ethics Committee

The study will be started at the institute only after approval from the IEC/IRB.

## Investigator Qualification

Any physician who is assigned PI of a clinical study must have professional integrity, must be familiar with the requirements of clinical studies and must be trained and experienced in the performance of clinical studies. A curriculum vitae*,* outlining the Investigator’s educational and professional qualification, has to be provided to the sponsor.

## Clinical Supplies and Storage

The Investigator will be provided with adequate quantities of the following material to permit completion of the study:

1. IB
2. Latest version of the study Protocol
3. CRFs
4. ICFs
5. IPs

The Investigator should maintain adequate records of the receipt and disposal of all IP supplied. Any unused medication will be returned to SIRO at the conclusion of the study. The Investigator should ensure that the IP is stored in a limited access area and protected from extremes of light, temperature and humidity.

## Data Collection, Documentation and Archiving of Study Documents

The required data have to be recorded in the CRF by the Investigator in anonymous form. Data listings must be generated from the original data of the CRF. In the medical report, it should be noted that the patient is participating in a clinical study and the study code and IP must be listed. All data and information on the study patient, laboratory values, and efficacy parameters have to be documented in the source data by the PI or designee. Patient data in the CRFs are coded in a way to enable easy tracking of the original patient data.

The CRF records must be complete and legible. The entries must be done using a black ink ballpoint pen. Any correction and modification must be entered, initialed and dated by staff authorized by the Investigator. Incorrect entries are not allowed to be obliterated by using e.g. Tipp-Ex^®^, eraser, white ink etc. Errors must remain readable.

**Patient and Medication Logs**

All patients coming to the investigational centre for the study indication during the course of the study will be recorded and numbered in a patient log. Only those patients, who fulfill the eligibility criteria, will be included in the study.

The Investigator is obliged to list all deliveries of IP in a medication log, thus documenting all deliveries of the sponsor and medication administered to the patients. The SIRO monitor will account for all used and unused drug supplies during the periodic monitoring visits, and supplies, as appropriate, would be retrieved. After termination of the study, samples, which have not been used, and the medication log must be returned to SIRO Clinpharm Pvt. Ltd. for control counting.

## Study Initiation

After the IEC approval is available, a “Site Initiation Visit” will be held before the first patient is enrolled on the study. The patients cannot be recruited until occurrence of such visit and its documentation. During this visit requirements of GCP, protocol procedures and all logistic issues will be discussed at length. The training of Investigator team will be documented.

## Study Monitoring and Source Data Verification

After the study is initiated, SIRO Clinpharm Pvt. Ltd. monitor(s) will take up contact with the Investigator to obtain information on the performance of the study. These contacts will be scheduled to take place at regular intervals and be either in person or telephonic.

These monitoring visits will take place at least 9 times during the entire study. The monitoring visits will be with an interval of 4-6 weeks followed by a close out visit at the end of the study.

Subsequent to start of recruitment, the first ***“*Routine Monitoring Visit”** would occur as soon as possible after recruitment of the first patient.

**Subsequent 2 Visits** would generally occur at an interval of 4 weeks while the **remaining visits** would occur at an interval of 4-6 weeks till end of study (after prior appointment with the Investigators).

The Investigator and his/her staff are obliged to set aside adequate time and place for the monitoring visits. During each monitoring visit, the monitor will review the CRFs of each patient in the study with regard to completeness, thoroughness and compliance to the protocol.

All hospital records/other records where original entries are made will be reviewed to make sure that-

1. Patient informed consent is incorporated
2. Inclusion/exclusion criteria are properly fulfilled
3. The CRF data are consistent with the physician's original records, which also have to clearly indicate that the patient is included in a clinical study
4. All relevant clinical and laboratory findings and concomitant medication are documented in the CRFs
5. Quantity and dosing schedule of concomitant medication is documented in the CRF
6. Quantity and dosing schedule of the IP/Comparator Product is in accordance with the protocol
7. All relevant information (e.g. any AE) has been recorded in the appropriate place in the CRFs
8. The IP/Comparator Product is being stored correctly, and its supply being properly accounted for
9. Incorrect or illegible entries in the CRFs would be submitted to the Investigator for correction.

The SIRO monitor will retrieve completed CRFs during the regularly held monitoring visits.

During the entire course of the study the responsible SIRO staff will be available to answer questions with regard to the performance of the study.

## Record Retention

All documentation pertaining to the study will be kept by Bharat Serums and Vaccines Ltd. for the lifetime of the product. The final report pertaining to this study will be kept by Bharat Serums and Vaccines Ltd. for a further five years.

The study monitor will provide each Investigator with a Site Master File*,* which should be used to file the IB, protocol, drug accountability records, correspondence with the IRB/IEC, Bharat Serums and Vaccines Ltd., SIRO and other study-related documents.

As required by ICH GCP guidelines, the Investigator must keep essential documents until at least 2 years after the last approval of a marketing application in an ICH region, and until there are no pending or contemplated marketing applications in an ICH region, or at least 2 years have elapsed since the formal discontinuation of clinical development of the IP.

These documents should be retained for a longer period, however, if required by the applicable regulatory requirement*(s)* or if needed by the Sponsor. In addition, the Investigator must make provision for the patient’s medical records to be kept for the same period of time.

No data should be destroyed without the agreement of the Sponsor Bharat Serums and Vaccines Ltd. will inform the Investigator in writing of the need for record retention and will notify the Investigator in writing when the trial related records are no longer needed.

Patients’ medical records and other original data will be archived in accordance with the archiving regulations or facilities of the investigational sites.

## Insurance and Indemnity

In the event that a patient suffers injury or death directly attributable to participation in this study, appropriate treatment and/or compensation will be provided by and/or paid to the patient by the Sponsor in accordance with applicable national laws and/or guidelines.

## Audits and Inspection

In addition to the above outlined monitoring visits, the investigational centres may be audited or inspected. The audit may be carried out by the Quality Assurance Department of SIRO Clinpharm Pvt. Ltd. or Sponsor *(Bharat Serums and Vaccines Ltd.)* or subcontracted by the Sponsor. In addition, regulatory authorities may inspect the study. Patient confidentiality will be maintained at all times to the extent permitted by the law. Audits and Inspections are conducted to assess compliance to the protocol, SOPs, ICH guidelines, regulatory requirements and any applicable guidelines.

The Investigator undertakes to expeditiously inform SIRO Clinpharm Pvt. Ltd. of an inspection requested by a regulatory authority.

## Data Management and Archival

Data management will be performed at SIRO Clinpharm Pvt. Ltd. according to SIRO SOPs. Data items from the CRFs will be entered centrally into the study database by CDM Staff using double data entry with interactive verification upon second entry ensure accurate data entry. Data reviewers & Medical Reviewers will perform a manual review of CRF pages as well as data discrepancies generated by electronic edit check programs. Any discrepancies noted in the data will be sent to site for resolution as data query forms. After all the data queries are resolved the database will be declared to be complete and accurate and will be locked for final analysis.

Copies of CRFs, other original source data related to the study, the site master file (which would contain, among other documents, the Patient Log and ICFs) would be archived until the sponsor requires them to be archived. The sponsor would inform the Investigator when the documents need no longer be retained at the study site.

## Patient Confidentiality and Data Protection

SIRO and Bharat Serums and Vaccines Ltd. will affirm and uphold the principle of the patient’s right to protection against the invasion of privacy. Throughout this study and any subsequent data analyses, all data reported to the sponsor will be identified only by protocol number, patient number and patient initials.

## Final Study Report and Publication

SIRO Clinpharm Pvt. Ltd. shall make an integrated clinical-biometrics final report according to ICH ‑ GCP tripartite guidelines which, after mutual consent, will also be signed by the main Investigator.

Publication of the study is independent of analysis results. Any formal presentation or publication of study would be considered as a joint publication by the Investigator(s) and the respective SIRO personnel. Authorship will be determined by mutual agreement. Monitoring alone will not be considered as a sufficient criterion for a co-authorship.

In this respect SIRO Clinpharm Pvt Ltd request that they receive copies of any intended publication early enough (at least 15 work days for an abstract or an oral presentation, at least 45 work days for a manuscript*)* to confirm the following aspects:

- Accuracy of data and findings *(*to avoid potential discrepancies with submissions to regulatory authorities)
- To prevent confidential information being published in error,
- To give the Investigator supplementary information of which he had been unaware
- To clarify the question of co-authorship.

# 23. REFERENCES

1. Bennet JE. Antimicrobial Agents (Continued): Antigungal Agents. In; *Goodman and Gillman’s*The pharmacological Basis of Therapeutics, 10^th^ ed. (Hardman J.G., Limbird LEGilman A.G., eds.) McGraw-Hill Companies Inc., New York, 2001, pp 1295 – 1312 (Simple mixture with lipid emulsions not enough).
2. Chandra J, Anand V, Patwari AK, et al. Kala-azar: experience from a non-endemic area in India. J Trop Pediatr (England), Oct 1995, 41(5) p298-300.
3. Gupta J, Balasegaram M, Sinha PK et al. Efficacy and safety outcomes of liposomal amphotericin B (AmBisome^®^) treatment for Indian visceral leishmaniasis under routine programme conditions in Bihar, India. MSF Scientific Day 2008. <http://www.msf.org.uk/UploadedFiles/3_KalaAzar_and_Ambisome_Abstract_FINAL_200806110343.pdf> (last accessed on 08 Sep 2008)
4. Sundar S, Gupta LB, Rastogi V, et al. Short-course, cost-effective treatment with Amphotericin B-fat emulsion cures Visceral Leishmaniasis. Trans R Soc Trop Med Hyg (England), Mar-Apr 2000, 94(2) p200-4 (Cost of lipid formulations).
5. Sundar S, Jha TK, Thakur CP, Mishra M, Singh VR, Buffels R. Low-dose liposomal amphotericin B in refractory Indian Visceral Leishmaniasis: a multicenter study. Am J Trop Med Hyg 2002; 66: 143-6.
6. Tabosa Do Egito, E. S., Appel, M., Fessi, H., Barratt, G., Puisieux, F., and Devissaguet, J.-P. In-vitro and in-vivo evaluation of a new amphotericin B emulsion-based delivery system. J Antimicrob Chemother 1996; 38: 485-497.
7. Thakur CP, Sinha GP, Pandey AK, et al. Do the diminishing efficacy and increasing toxicity of sodium stibogluconate in the treatment of Visceral Leishmaniasis in Bihar, India; justify its continued use as a first-line drug? An observational study of 80 cases. Ann Trop Med Parasitol (England), Jul 1998, 92(5) p561-9.
8. Thakur CP, Sinha GP, Sharma V, et al. Evaluation of Amphotericin B as a first line drug in comparison to sodium stibogluconate in the treatment of fresh cases of Kala-azar. Indian J Med Res (India), Jul 1993, 97 p170-5.
9. Thakur CP. A single high dose treatment of Kala-azar with AmBisome (Amphotericin B lipid complex): a pilot study. Int J Antimicrob Agents 2001 Jan;17(1):67-70.
10. Thakur CP., Leishmaniasis. In; API textbook of Medicine, 6th ed. (Sainani GS., ed). Association of Physicians of India, Mumbai 1999, pp 92-95.
